# Matrimid-JUC-62 and Matrimid-PCN-250 mixed matrix membranes displaying light-responsive gas separation and beneficial ageing characteristics for CO_2_/N_2_ separation

**DOI:** 10.1038/s41598-018-21263-7

**Published:** 2018-02-13

**Authors:** Nicholaus Prasetya, Anastasia A. Teck, Bradley P. Ladewig

**Affiliations:** Barrer Centre, Department of Chemical Engineering, Imperial College London, Exhibition Road, London, SW7 2AZ United Kingdom

## Abstract

The performance of two generation-3 light-responsive metal-organic framework (MOF), namely JUC-62 and PCN-250, was investigated in a mixed matrix membrane (MMM) form. Both of them were incorporated inside the matrimid as the polymer matrix. Using our custom-designed membrane testing cell, it was observed that the MMMs showed up to 9% difference in CO_2_ permeability between its pristine and UV-irradiated condition. This shows that the light-responsive ability of the light-responsive MOFs could still be maintained. Thus, this finding is applicable in designing a smart material. Apart from that, the MMMs also has the potential to be applied for post-combustion carbon capture. At loadings up to 15 wt%, both CO_2_ permeability and CO_2_/N_2_ ideal selectivity could be significantly improved and surpassed the value exhibited by most of the MOF-matrimid MMM. Lastly the long term performance of the MMM was also evaluated and it was observed that both MMM could maintain their performance up to 1 month with only a slight decrease in CO_2_ permeability observed for 10 wt% PCN-250-matrimid. This study then opens up the possibility to fabricate a novel anti-aging multifunctional membrane material that is applicable as a smart material and also in post combustion carbon capture applications.

## Introduction

Membranes have been widely investigated for various promising applications ranging from energy-related application to smart material^1,2^. In many membrane applications, polymeric membranes are still widely used because of their ease of processing and economical considerations. There are then various ways to modify and improve the properties of the pristine polymeric membrane that is limited by its own property. Mixed-matrix approach is a promising way to accomplish the task because of its simplicity in processing. In mixed matrix membrane, functionalized materials are usually incorporated inside the polymer membrane to improve the overall mixed matrix membranes property. Various functionalized materials can then be used to fabricate mixed matrix membranes such as: carbon nanotube^3,4^, zeolite^5,6^ and recently metal-organic framework (MOF)^7–9^.

Metal-organic framework (MOF) is a relatively new class of hybrid material built from metal cluster linked by organic ligands resulting in a porous structure. MOFs have gained increased interest because of various reasons starting from their exceptional physical properties such as large pore size, high surface area, pore size tailorability and their promising applications in gas separation, catalysis, template for constructing other materials, and other applications^10–13^. Recent developments have also shown the possibility to render MOFs with a stimuli-responsive ability. Such MOFs can then exhibit different behaviours upon exposure to different circumstances such as: the presence of guest molecules^14^, changing in pressure^15^, temperature fluctuation^16^, magnetic condition^17^, and light exposure^18,19^. Among the stimulants, light can be considered as the most convenient because of its abundance and rarely generate any side products.

This then leads to developments in various light-responsive MOFs that can be further divided into three main categories: generation 1 with photoswitchable guest molecule^20,21^, generation-2 that has pendant photoswitchable moiety inside the pore^22–25^ and generation-3 whose framework is built using a photoresponsive ligand^26–29^. Such MOF can be tailored to have different behaviour upon exposure to UV light or visible light. In particular, such behavioural difference is usually observed by different CO_2_ adsorption, which makes them also applicable for low energy CO_2_ capture^30^. Various studies have been conducted since then using light-responsive MOFs when they were fabricated as a MOF membrane and tested their performance^31–34^. However, to the best of our knowledge, there is no research up to now studying its performance once they are incorporated inside a polymer matrix to form a mixed matrix membrane and in particular to address the challenge to have a multifunctional material.

This study then focuses on studying the performance of generation-3 light-responsive MOF in a mixed matrix membrane to improve the overall property of the resulting mixed matrix membranes. Generation-3 light-responsive MOFs were chosen because their pores are not obstructed by any pendant group of the light-responsive moiety. This condition is preferable to maximize the porosity in the MOFs which also contributes to enhancing the mixed matrix membrane performance. Apart from that, generation-3 light-responsive MOFs are also known to be efficient for photo-switching ability, since they have instantaneous response under different light conditions. In particular for this study, JUC-62 and PCN-250 were chosen as the light-responsive MOFs since they are relatively stable when stored under ambient condition. Apart from that, we are interested in this topic because the wider applicability of such light-responsive MOFs has been rarely explored.

Thus say, this study focuses on two different areas where these mixed matrix membranes can be applied. Firstly, the light-responsive ability of the resulting mixed matrix membranes were evaluated. This importance of the study lies once such membranes are to be applied in an area that requires stimuli-responsive materials such as microcontroller or smart-membrane area. Secondly, we studied the mixed matrix membranes for the post-combustion carbon capture area for CO_2_/N_2_ separation. This is because various studies have shown improvement in gas separation property coming from mixed matrix membranes^7^. To complete the study, we also investigated the aging of the mixed matrix membranes to evaluate their long-term performance. This study will then contribute in designing a platform for a multifunctional membrane that is applicable in smart material and clean energy.

## Results

### Mixed matrix membrane characterization

As stated above, JUC-62 and PCN-250 can be classified as ones of the most recent generation-3 light-responsive MOFs since they are built from a responsive framework. The crystal structure of these two materials can be seen in Figures S3 and S4 from supplementary information. The BET surface area for both light-responsive MOFs calculated from nitrogen sorption data was found to be around 1020 and 1376 m^2^/g, respectively. Both JUC-62 and PCN-250-matrimid mixed matrix membranes were then characterized by using various characterization techniques such as PXRD, FTIR, TGA and SEM.

**Figure 1:**
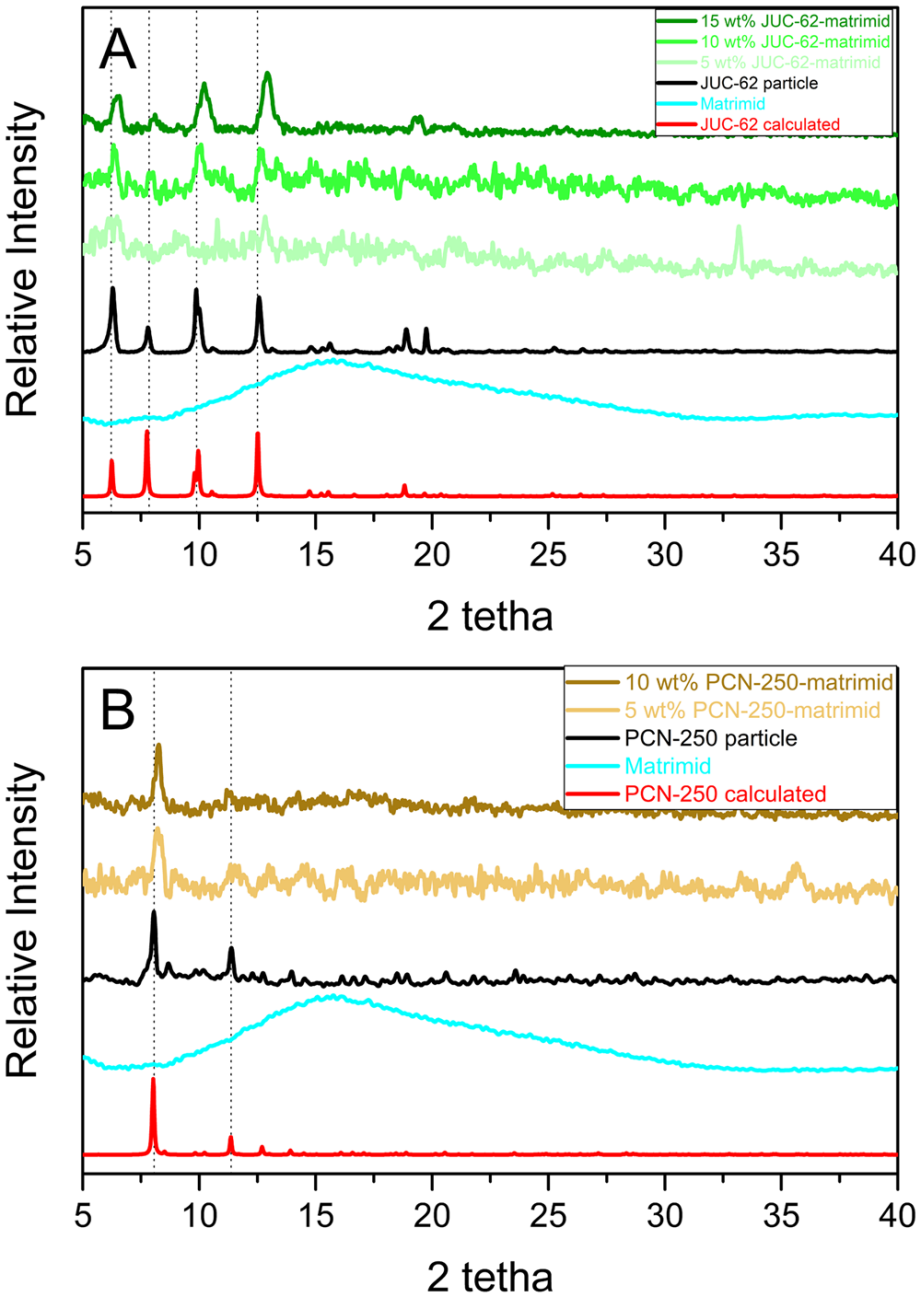
PXRD Spectrum of JUC-62 (**A**) and PCN-250 (**B**) matrimid mixed matrix membrane.

Figure 1 shows the PXRD spectrum of the calculated pattern of the MOFs, MOFs particle, matrimid and the mixed-matrix membranes. Matrimid has a broad PXRD spectrum indicating its nature as a non-crystalline material. It could be seen that the PXRD spectrum of the resulting mixed matrix membranes started to give identity peaks of the MOFs at a relatively low loading. The relative intensity of the MOFs’ identity peaks started to increase once the loading got higher. For JUC-62-matrimid mixed matrix membranes, all 4 identity peaks of JUC-62 below 20° clearly appeared at 15 wt% loading while the 10 wt% PCN-250-matrimid mixed matrix membrane gave two identity peaks owned by PCN-250 particle. It could also be observed that compared to the MOF particles and its calculated pattern, all the peaks in the mixed matrix membrane were slightly shifted toward higher 2θ value. It has been previously observed that a shift in PXRD pattern of a MOF-mixed matrix membrane could actually be shifted because of the interaction between the polymer and the framework^35^. According to Bragg’s Law, increase in 2θ value corresponds to the decrease in lattice parameter. In our case, the interaction occurs between the matrimid and the MOF’s framework, which can come from the azobenene group, might result in a slight contraction of the MOF framework^36,37^. However, this does not destroy the MOF framework since all the identity peaks could still be preserved.

**Figure 2:**
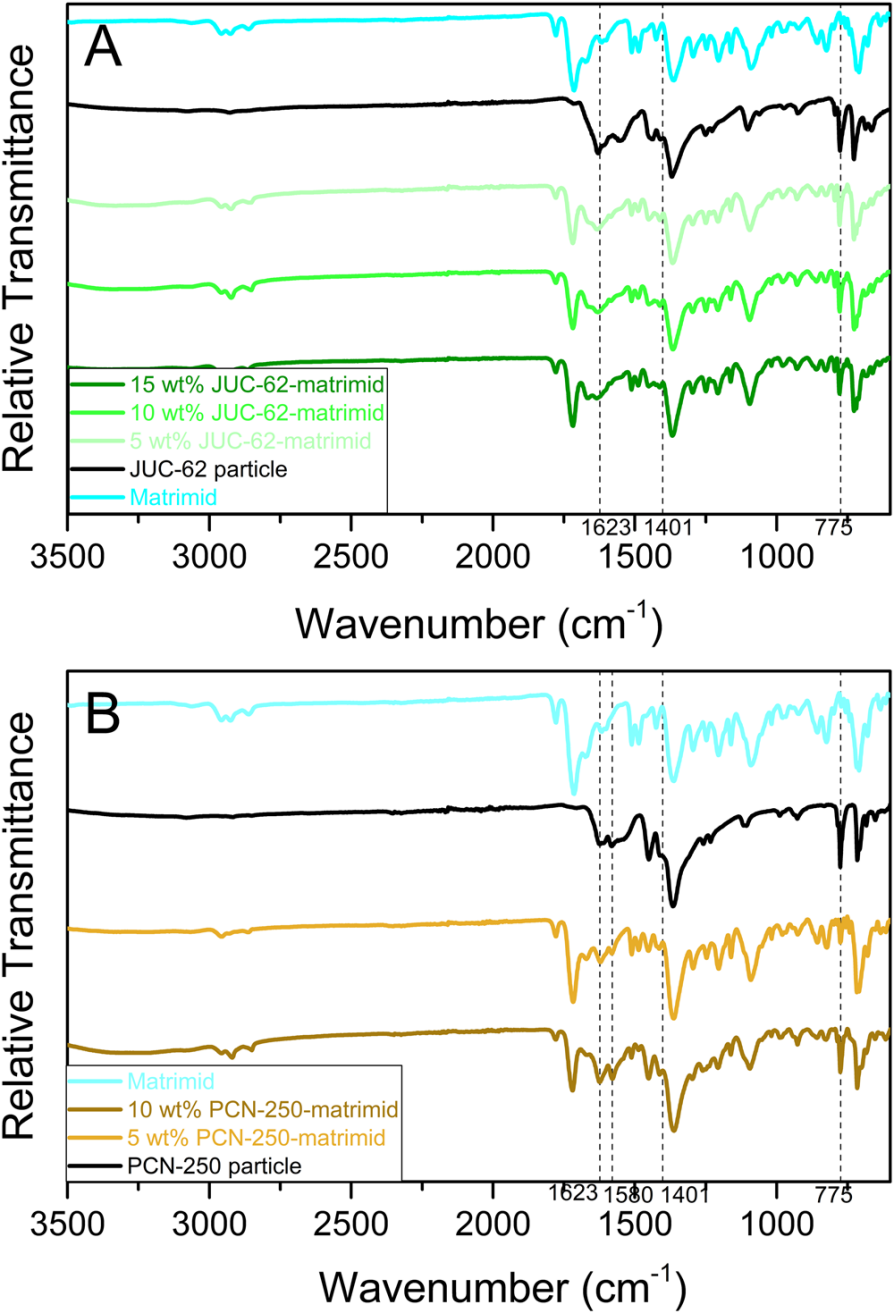
FTIR Spectrum of JUC-62 (**A**) and PCN-250 (**B**) matrimid mixed matrix membrane.

Figure 2 shows the FTIR spectrum of the MOFs, bare matrimid and the mixed matrix membranes. In general, all the mixed matrix membranes spectrums have additional peaks that appear around 1620, 1400, and 770 cm^−1^ which are characteristic to the presence of azobenzene group^38–40^. In particular, the “fingerprint” region between 1700–1000 cm^−1^ could be attributed to the vibration of C=C from azo-phenyl group that occurs around 1600 cm^−1^ ^39^ and N=N bonding coming from the azobenzene group at around 1400 cm^−1^ ^40^. These then give an indication of the presence of the light-responsive MOFs inside the mixed matrix membranes.

**Figure 3:**
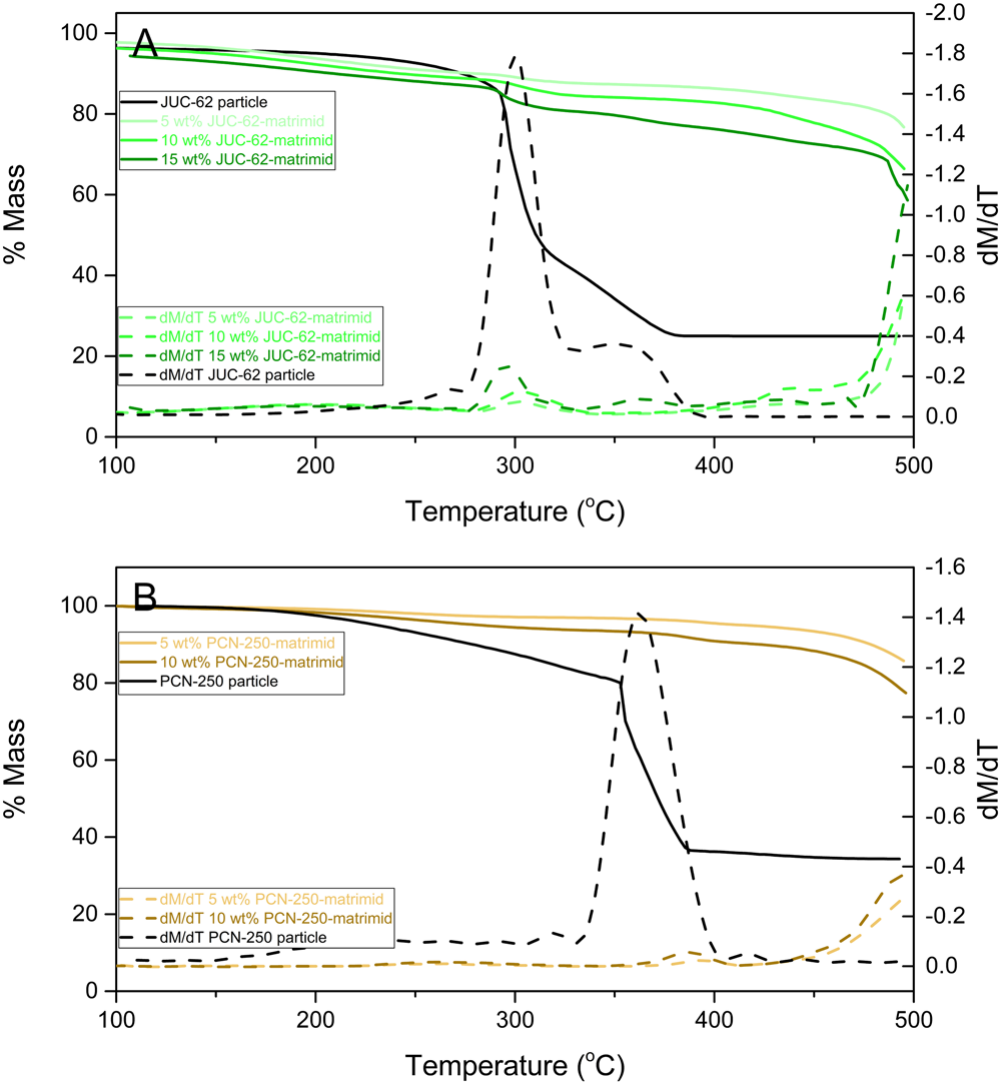
TGA data analysis of JUC-62 (**A**) and PCN-250 (**B**) matrimid mixed matrix membrane.

Thermal analysis was also performed on the mixed matrix membranes. The TGA data is shown in Fig. 3 and shows the agreement between the mass loss inside the matrix and the mass loss from the pure MOF. Between 250 °C–400 °C, JUC-62 lost about 70% of its total mass. It was found from TGA analysis that 5 wt%, 10 wt% and 15 wt% JUC-62-matrimid lost about 4%, 7% and 11% of its total mass, respectively. Meanwhile for PCN-250, the particle lost about 60% of its total mass after total degradation (between 250 °C–400 °C). And it was found that the mixed matrix membranes lost about 3% and 6% of its total mass for 5 wt% and 10 wt% PCN-250-matrimid, respectively.

It should be noted that the DSC analysis might not be suitable to be applied here. Usually in DSC analysis, a shift in glass transition temperature of the mixed matrix membranes should be expected. However, for the MOFs used in this study, the degradation of the framework was observed to start at around 300 °C where JUC-62 and PCN-250 underwent total and partial degradation, respectively. This temperature is below the glass transition temperature of matrimid which is reported to be around 320 °C. Thus say, a DSC analysis might not be too suitable to be applied here since before reaching the Tg of the matrimid, the MOFs inside the framework has already started to degrade which might not be able to give any impact on the mixed matrix membranes.

**Figure 4:**
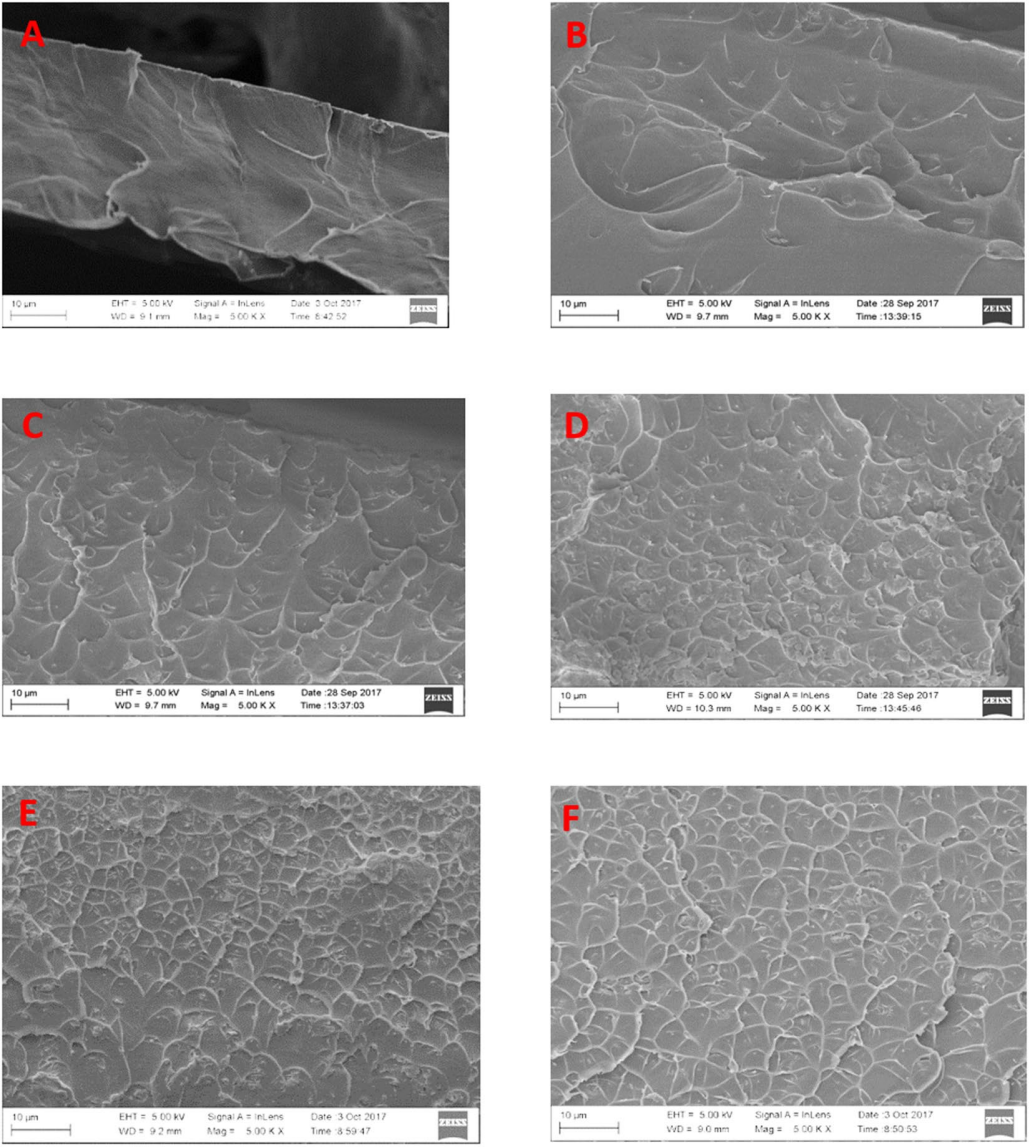
SEM picture of matrimid (**A**), 5 wt% JUC-62-matrimid (**B**), 10 wt% JUC-62-matrimid (**C**), 15 wt% JUC-62-matrimid (**D**), 5 wt% PCN-250-matrimid (**E**) and 10 wt% PCN-250-matrimid (**F**).

The dispersion of the MOFs particle inside the polymer matrix was analysed through microscopic image. As can be seen in Fig. 4, the MOFs particles could be distributed evenly inside the polymer matrix without any sign of defective interaction between the particle and polymer matrix. This qualitatively shows that both light-responsive MOFs have good interaction with the polymer.

Three different performance aspects were then evaluated on the resulting mixed-matrix membranes: light-responsive ability, CO_2_/N_2_ gas separation and long-term performance. Each of this will be discussed in more details below.

### Light-responsive against CO_2_ performance

It has been proven previously that both JUC-62^29^ and PCN-250^41^ are responsive against UV-light. Their CO_2_ adsorption capacity was observed to significantly decrease upon exposure to UV light^29,41^. This renders them as a candidate to be applied in low-energy CO_2_ capture because it then only requires low energy during regeneration. We are then interested to further study this light-responsive behaviour once they are incorporated inside a glassy polymer matrix. A potential application for this is around the area of smart material and sensor that requires materials that are responsive towards their surroundings.

During the experiment, controlling temperature was crucial to get the correct reading because the presence of UV light inside the membrane test cell that generated significant amount of heat. Temperature consistency is crucial since it has been reported that the pristine matrimid membrane gas permeation and selectivity could be significantly affected by change in temperature^42^. This was done firstly by putting a thermocouple at the centre of the membrane test cell to continuously monitor the temperature consistency (Figure S11). Apart from that, we also did two different control experiments to ensure that the effect of the temperature was indeed negligible: pristine matrimid and 8 wt% ZIF-8-matrimid mixed matrix membrane (with no known photo responsive ligand).

**Figure 5:**
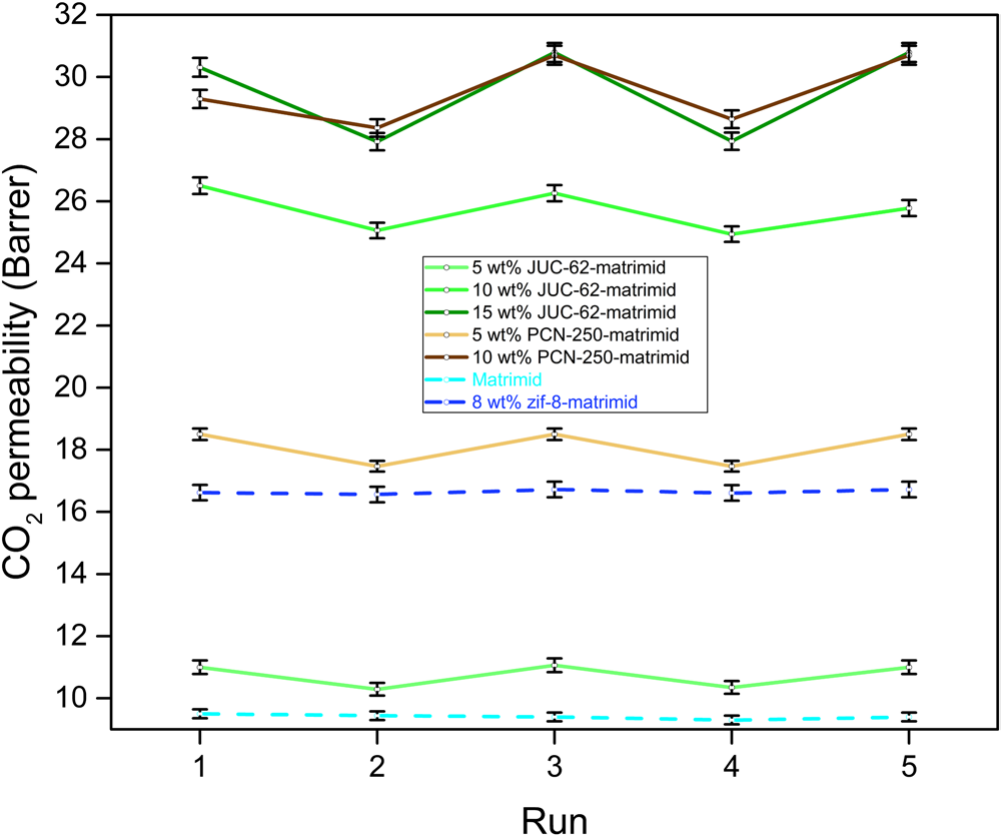
CO_2_ permeability light-responsive test.

It could be seen firstly from the Fig. 5 that the temperature effect was indeed negligible. There was almost neither an increase nor a decrease in CO_2_ permeation when the UV light was switched on. First of all, it could be seen that the permeability of matrimid could be well maintained to be around 9.5 ± 0.1 barrer, during the five cycles experiment with alternating switching of UV light. In addition to that, we did not observe any significant drop in matrimid permeability after it was irradiated with UV light as previously observed with the light-responsive PIM^43^. It might be caused by different wavelength used in this study. While this study used 365 nm UV wavelength, the previous one used 254 nm UV wavelength. It is then highly likely that the matrimid absorbs the wavelength in the 254 nm region resulting in partial densification thus resulting in reduced gas permeability. The negligible loss of permeability exhibited by matrimid studied under this particular wavelength was then quite important to later observe the switching phenomenon in the mixed matrix membrane.

This was further proven by another control experiment using 8 wt% ZIF-8-matrimid mixed matrix membrane. This importance of this control experiment was to observe the behaviour of a mixed matrix membrane that was loaded with MOF that does not have any light-responsive ability. As can be seen, a similar trend as observed in matrimid was also observed with the ZIF-8-matrimid mixed matrix membrane. It did not exhibit any difference regarding CO_2_ permeability during the five cycles alternating switching. The permeability could be consistently maintained to be around 16.6 ± 0.1 barrer during the alternating switching of UV light. Meanwhile, a slightly higher value in CO_2_ permeability might indicate the efficacy of incorporating ZIF-8 in a mixed matrix membrane as previously observed in ZIF-8 mixed matrix membranes^44^. Both control experiments then show the reliability of our home-made membrane testing cell and temperature difference during the alternating cycle was indeed negligible to alter the membrane CO_2_ permeability during the experiment.

However, a rather different trend was observed once light-responsive MOFs were incorporated inside the matrimid. As can be seen from Fig. 5, the resulting light-responsive MOFs mixed matrix membrane was observed to have a switching ability against CO_2_ while almost no switching observed for the nitrogen permeation (see Figure S13). In general, the CO_2_ permeability for all the light-responsive MOFs mixed matrix membrane was found to be lower compared when the UV light was switched on.

Table 1 then shows the extent of switching behaviour observed in the light-responsive mixed matrix membrane. It was observed that on average, the mixed matrix membranes gave around 5% difference in CO_2_ permeability. Although only a modest difference, such a behaviour was actually not observed from the previous control experiment which only exhibited around 1% different in CO_2_ permeability. Thus say, it could be safely inferred that the behaviour actually comes from the light-responsive MOFs imparted inside the polymer matrix. Moreover, it was observed that the highest switching degree was around 8.5% obtained in 15 wt% JUC-62-matrimid. This is probably because 15 wt% JUC-62-matrimid is the highest-loaded mixed matrix membranes used in this study and thus the extent of photoswitching in CO_2_ permeability was more pronounced than the rest of the membranes.

**Table 1:** CO_2_ permeability of light-responsive mixed matrix membranes with and without UV.

Membrane	Average CO_2_ permeability (±0.1 Barrer)		% change
	UV off	UV on	
Matrimid	9.5	9.4	1.05
8 wt% ZIF-8-matrimid	16.6	16.5	0.6
5 wt% JUC-62-matrimid	11	10.3	6.36
10 wt% JUC-62-matrimid	26.3	25	4.94
15 wt% JUC-62-matrimid	30.5	27.9	8.52
5 wt% PCN-250-matrimid	18.5	17.4	5.94
10 wt% PCN-250-matrimid	29.8	28.5	5.03

The result trend also reveals that the CO_2_ permeability trend does also agree with the behaviour of the light-responsive MOFs particle. Both JUC-62 and PCN-250^45^ have lower CO_2_ adsorption when they are irradiated with UV light than at their normal condition. Therefore, when the UV light inside the membrane cell was switched on, it could also be expected that the incorporated light-responsive MOFs inside the matrimid had lower interaction towards CO_2_ than when the UV light was switched off. As a consequence, lower CO_2_ permeation than when the UV light was off should also be expected.

In spite of the fact that the light-responsive MOF mixed matrix membrane showed lower CO_2_ permeability under UV light condition, the difference was somewhat lower than expected. From our investigation of CO_2_ and N_2_ permeability (discussed in the next section), the resulting light-responsive MOF mixed matrix membrane has shown its efficacy once incorporated inside the polymer matrix. This led us to think that the incorporated MOFs should have been able to contribute significantly to improve the overall transport properties inside the mixed matrix membrane. Therefore, we also expected a higher degree of switching properties of the mixed matrix membrane since in its pristine condition -when not embedded as an incorporated particle,- both MOFs were reported to have up to 50% decrease in CO_2_ uptake under the irradiation of UV light. However, about 8.5% was the highest switchable degree that we could obtain.

The most possible reason for this phenomenon is probably because of the rigid structure of the matrimid. Both JUC-62 and PCN-250 are classified as generation-3 light-responsive MOFs. It means that they are constructed from a light-responsive pillaring ligand for the framework. For the generation-3 light-responsive MOFs, ligand bending was found to be important in producing the ‘squeezing’ effect resulting in release of adsorbed CO_2_ from the adsorbent. It is very likely that this ligand bending phenomenon was limited when glassy polymer like matrimid was used as the polymer matrix. As a result, a very significant change in CO_2_ permeability could not be observed when the UV light was switched on. This could also be the reason that we only observed a slight difference in FTIR spectrum between the non-irradiated and irradiated light-responsive mixed matrix membranes in the 600–500 cm^−1^ region (Figure S12 in supplementary information) as opposed to significant difference that was previously observed for the light-responsive MOFs^28,29,41^. A similar phenomenon has also been observed previously using Mil-53 (Al) in matrimid matrix. The authors also argue that the matrimid could rigidly maintain the pore opening of the Mil-53 (Al) framework which was known to be breathable^46^. Such pore maintenance in by glassy polymer matrix may then hinder to some extent the ‘squeezing’ of the light-responsive MOFs that is responsible for delivering the light-responsive ability of the generation-3 light-responsive MOFs.

### CO_2_/N_2_ separation performance

Apart from the light-responsive ability of the resulting mixed matrix membranes, the mixed matrix membranes performance were also evaluated regarding its CO_2_/N_2_ separation performance. This performance evaluation is important when the mixed matrix membrane is to be considered to be applied in post-combustion carbon capture. Table 2 then gives the summary of the permeability of CO_2_ and N_2_ for the resulting mixed matrix membranes used in this study.

**Table 2:** Permeability and CO_2_/N_2_ ideal selectivity for the membranes used in this study.

Membrane	Permeability (Barrer)		Ideal Selectivity (CO_2_/N_2_)
	CO_2_	N_2_	
Matrimid	9.5	0.29	32.7
8 wt% ZIF-8-matrimid	16.6	0.48	34.6
5 wt% JUC-62-matrimid	11	0.29	37.9
10 wt% JUC-62-matrimid	26.3	0.65	40.5
15 wt% JUC-62-matrimid	30.5	0.54	56.5
5 wt% PCN-250-matrimid	18.5	0.51	37
10 wt% PCN-250-matrimid	29.5	0.46	64.1

**Figure 6:**
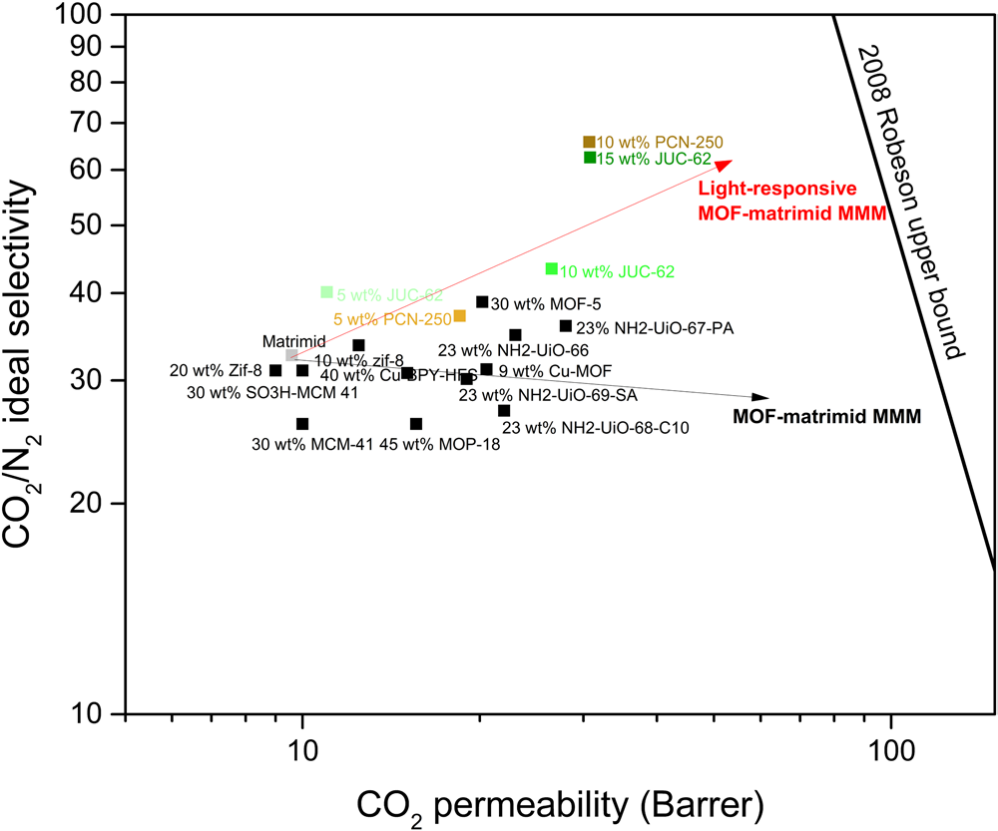
CO_2_/N_2_ separation performance of light-responsive MOF-Matrimid mixed matrix membrane.

It could be clearly seen that incorporating the light-responsive MOFs inside matrimid (both JUC-62 and PCN-250) renders a positive impact to the overall membrane performance. The CO_2_ permeability could be increased up to 100% for the mixed matrix membranes with the highest particle loading. This positive effect is actually expected since the incorporated MOFs should give additional pathway for gases to permeate through the membranes. Furthermore, it could also be seen that PCN-250 gives higher increase in CO_2_ permeability in comparison to JUC-62. The loading of the JUC-62-matrimid had to be around 15 wt% to have a comparable permeability performance with the 10 wt% PCN-250-matrimid. The reason for this is probably because JUC-62 has lower CO_2_ adsorption capacity compared with PCN-250. At 1 bar and 273 K, the CO_2_ adsorption capacity of PCN-250 is found to be 146 cm^3^/g while JUC-62 only has 107 cm^3^/g STP. Lower adsorption capacity in JUC-62 than PCN-250 would probably mean that PCN-250 has more active sites for CO_2_ to permeate through once they were incorporated inside the mixed matrix membranes resulting in higher CO_2_ permeability than JUC-62 with the same loading.

Surprisingly, CO_2_ is not the only parameter which was positively affected. A positive increase was also observed in terms of CO_2_/N_2_ membrane selectivity. For the highest particle loading, this ideal selectivity could be increased up to 100% resulting in ideal selectivity around 60 both for 15 wt% JUC-62 and 10 wt% PCN-250-matrimid. This behaviour is actually somewhat unexpected.

A simulation study has been conducted previously showing that for most of the MOFs available in literature and with using matrimid as the polymer, only CO_2_ permeability could be increased while a positive impact in CO_2_/N_2_ selectivity could not be expected^47^. This is mainly attributed to the fact that almost all MOFs have significantly higher permeability than any polymer matrix and thus able to improve CO_2_ permeability. But this has also to be compensated by increase in nitrogen permeability and thus resulting in barely improved selectivity. As can be seen from Fig. 6, this simulation does seem to be true for most of the MOFs studied in real experiment^48–53^. Most of the MOFs-matrimid mixed matrix membrane studied so far has only shown an increase in CO_2_ permeability while barely gave any positive impact on ideal selectivity. Our study with 8 wt% ZIF-8-matrimid that we used for control experiment in light-responsive study also showed barely improvement in selectivity while its CO_2_ permeability could be increased. There are some exceptions, however. For instance, some MOFs like the UiO family has been reported before to be able to increase the ideal selectivity once incorporated inside the matrimid with 23 wt% loading^50^. In spite of this fact, the ideal selectivity improvement reported was only around 25%.

In contrast to all of these atomistic studies and experimental findings, our mixed matrix membrane has surprisingly shown an increase in ideal selectivity of up to 100%. This value is then probably one of the highest reported so far in the area of MOFs-matrimid mixed matrix membrane for CO_2_/N_2_ separation. In order to make a fairer comparison, Figure S14 in supplementary information then gives the plot of CO_2_/N_2_ ideal selectivity reported in the literature for MOF-matrimid dense mixed matrix membrane system in comparison with their pristine matrimid measured at their operating condition. By observing the plot, it could be safely inferred that our CO_2_/N_2_ ideal selectivity increase could not be caused by the intrinsic property of the matrimid used in this study or because of the operating condition. Rather, it came from the MOF incorporated inside the matrimid.

Apart from good interaction and dispersion of the MOF particle inside the polymer matrix as observed in SEM, this phenomenon could also be attributed to the presence of azobenzene functionality inside the light-responsive MOFs. Various investigations have been made previously about the benefit of having azo-functionality for CO_2_/N_2_ separation in the area of porous material. Various mechanisms do exist when bringing in this beneficial aspect. Azobenzene has been previously known to have a good affinity towards CO_2_ because of its Lewis acid-base interaction^54^ and dipole-quadrupole interaction^55^. This beneficial interaction may then enhance CO_2_ transport through the light-responsive matrimid mixed matrix membranes used in this study.

Furthermore, from the nitrogen transport point of view, Patel and co-workers recently coined the term of nitrogen phobic environment existing in the series of the azo porous organic framework that they synthesized^56,57^. Their careful analysis to the available azo-based porous framework has shown that bonding between N_2_ and the azo-group is less favourable than CO_2_ and the azo-group resulting in unprecedentedly high CO_2_/N_2_ selectivity. Such theory has also been proven with various azo-based porous organic polymers^58–60^. A similar investigation has also been conducted for MOF in particular^61^. In the study, it was observed that the MOF that contains azo linkage (-N=N-) has higher CO_2_/N_2_ selectivity than the MOF that has stilbene linkage (-CH=CH-). Although they only attributed this to the higher CO_2_ adsorption because of the polarity of the presence of azo-linkage, it should be noted as well that the azo-linked MOF exhibited lower nitrogen adsorption compared with its azo-linked counterpart. Thus, the presence of azo-group is beneficial both for improving CO_2_ affinity and reducing N_2_ uptake in the MOF framework. This then can also explain the higher selectivity observed in the mixed matrix membranes once azo-based MOF as used in this study are embedded inside a polymer matrix. This is proven by looking at the trend obtained for the nitrogen permeability for the mixed matrix membranes. As can be seen, less nitrogen permeability was observed as the loading in the mixed matrix membrane was increased. This is in contrast to the CO_2_ permeability that kept increasing with higher MOF loading. This condition then show that the mixed matrix membranes could attract more CO_2_ to permeate through the membrane while the same thing could not be expected for nitrogen permeation which could be attributed to the presence of azobenzene group.

In addition to that, achieving this exceptional performance did not require a very high loading mixed matrix membrane (between 10–15 wt%). Another advantage with our mixed matrix membrane is no modification to the MOF was required to obtain this high selectivity value. A combination of simple blending with polymer and with relatively low loading made these mixed matrix membrane ease in preparation. Thus say, our approach in fabricating these mixed matrix membranes do seem promising in post-combustion carbon capture application.

### Long term performance study

Lastly, the long term performance of the resulting mixed matrix membrane was also evaluated. We evaluated the long term performance both for 15 wt% JUC-62-matrimid and 10 wt% PCN-250-matrimid as these two membranes gave the best performance. After the first testing, the membranes was left in the ambient condition for about a month (2 months for the 15 wt% JUC-62-matrimid) before its performance were re-evaluated

**Figure 7:**
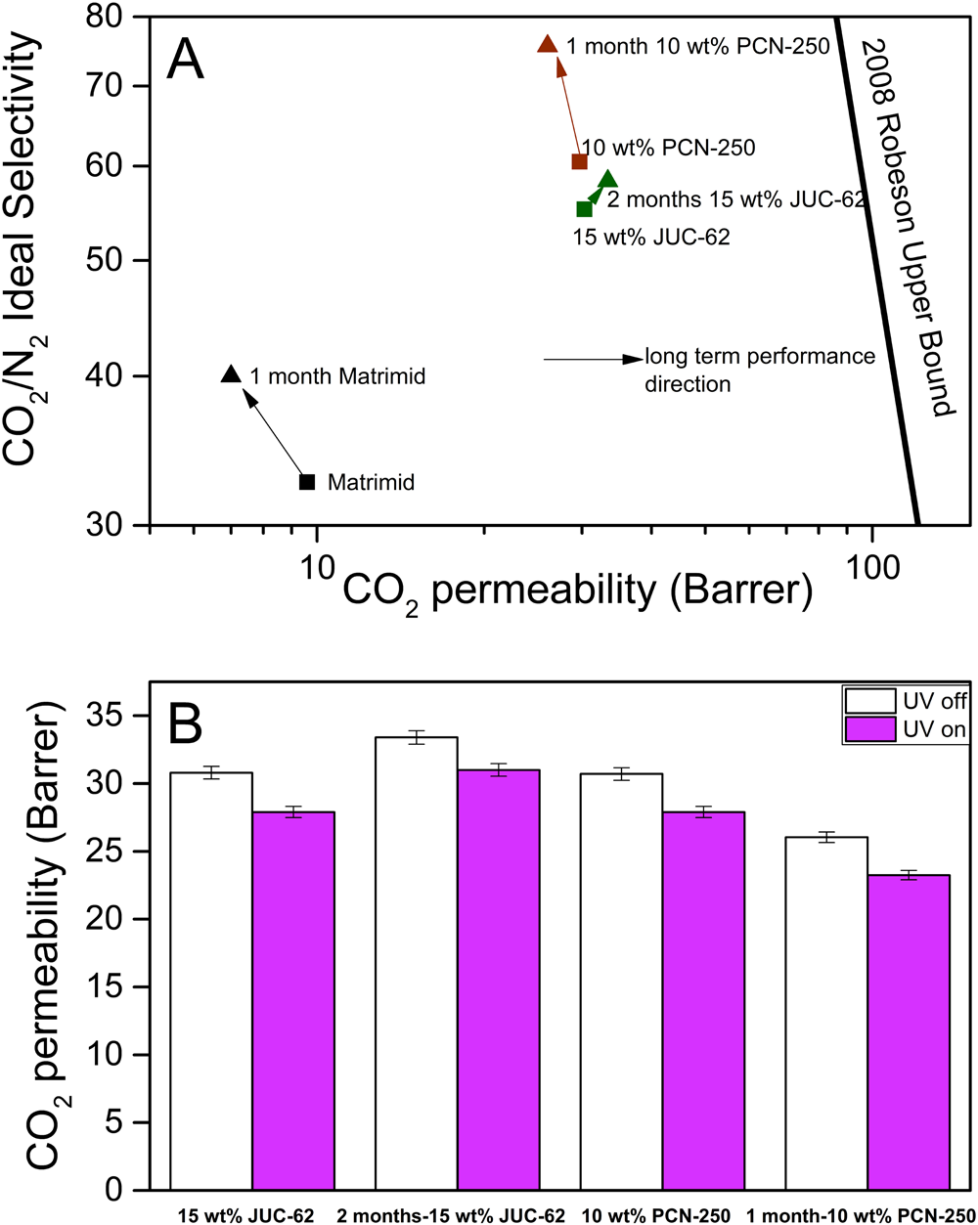
Long-term performance of the light-responsive mixed matrix membranes in terms of CO_2_/N_2_ ideal selectivity (**A**) and light-responsive CO_2_ permeability (**B**).

As can be seen from Fig. 7, the pure matrimid membrane experienced a quite severe aging. The permeability loss of the matrimid was found to be around 25% and it dropped from 9.5 barrer to be about 7 barrer. As usually observed in physical aging, the selectivity of the matrimid also went up from 32 to be around 40. This indicates the physical aging actually happened in the matrimid membrane. In physical aging, polymeric membrane undergoes structural change resulting lower free volume in the matrix. As a consequence, the permeability of the membrane drops and it is usually accompanied by increase in selectivity.

A rather contrast situation was observed for 10 wt% PCN-250-matrimid. Although a slight decrease in CO_2_ permeability was observed for the mixed matrix membranes, the change rate was not as pronounced as the matrimid membrane. The permeability dropped for about 10% from about 20 to be around 18 barrer. This was also accompanied by an increase in selectivity from 64 to 75. Thus say, the 10 wt% PCN-250-matrimid did seem to also age during the storage although it was not as pronounced as the pure matrimid membrane. It also indicates that incorporating PCN-250 did help to maintain the long term performance of the mixed matrix membrane. The light-responsive behaviour was also evaluated the aged PCN-250-matrimid membrane. As can also be seen in the Fig. 7 the aged mixed matrix membrane did still have lower permeability when it was irradiated with UV light.

Interestingly, in contrast to the matrimid and 10 wt% PCN-250-matrimid, the 15 wt% JUC-62-matrimid showed negligible loss in performance. Even after two months left at ambient condition, the performance of 15 wt% JUC-62-matrimid could still be preserved. The CO_2_ permeability were still around 20 barrer with CO_2_/N_2_ ideal selectivity found to be around 56. Previously, a similar phenomenon was also observed with PIM-1 and PTMSP added with nano-fillers^62,63^. They found that the mixed matrix membrane did not experience any decrease in permeability and even the selectivity was increased after storing at ambient condition for a long period of time. They attributed this to the good interaction that occurs between the PAF and PIM that prevents the PIM to undergo any structural change during storage. A good interaction could also be then established between the light-responsive MOF, especially JUC-62, and matrimid resulting in stability in long-term membrane performance. This might also partially explains why we only observed a rather limited light-responsive ability coming out from the light-responsive MOF. Such good interaction might probably limit the ability of the MOF framework to undergo bending when they were irradiated with UV light.

In terms for light-responsiveness, it could also be seen from Fig. 7 that both light-responsive MOF-matrimid MMM still gave a light-responsive ability. The 15 wt% JUC-62-matrimid and 10 wt% PCN-250-matrimid could still give around 8% and 6%, respectively, difference in CO_2_ permeability during the photo-switching experiment. This then corroborated the previous result for CO_2_/N_2_ separation performance showing that the MOFs were still intact inside the polymer system and its porosity could still be maintained resulting in a positive impact both on gas separation and light-responsive ability. This might be attributed to the relative stability of both of the MOFs as previously observed. Apart from the intrinsic property of the MOF stability, incorporation of MOFs within the matrimid matrix might also help to maintain the MOFs porosity. This factor might play a role can be judged from the previous result from the CO_2_/N_2_ separation where a good interaction between the two materials might be established and thus might help to improve the MOF overall stability.

In conclusion, light-responsive MOFs-matrimid mixed matrix membranes have been successfully fabricated using JUC-62 and PCN-250 as the light-responsive MOFs. Using our unique home-made membrane testing cell, it was observed that around 10% decrease in CO_2_ permeability could be observed when the mixed matrix membranes was irradiated with UV light. In spite of this fact, the rigid structure of matrimid might limit the light-responsive ability of the MOFs that depends on the flexibility of the framework. In terms of CO_2_/N_2_ separation performance, the resulting mixed matrix membranes exhibited a superior performance in comparison with MOFs-matrimid mixed matrix membranes fabricated so far. The ideal selectivity of the resulting mixed matrix membrane could be significantly improved. In particular, we observed up to 100% increase in ideal selectivity obtained with a relatively low loading of MOFs particle inside the matrimid (15 wt% for JUC-62 and 10 wt% for PCN-250). This renders them to be applicable for post-combustion carbon capture. Long-term performance showed that the performance only slightly decreased for the 10 wt% PCN-250-matrimid while it was negligible for 15 wt% JUC-62-matrimid. This shows that good interaction actually occurs between the polymer and the MOFs. This study then opens up the possibility in constructing a novel multi-functional anti-aging material that is photo-responsive for smart material while also demonstrate a highly effective separating performance of CO_2_/N_2_ which is beneficial for post combustion carbon capture.

## Methods

### Synthesis of azobenzene tetracarboxylic acid (H_4_ABTC)

H_4_ABTC was synthesized according to the previously reported method^64^. In a typical synthesis, 5 g of 5-nitroisopthalic acid was dissolved in an aqueous solution containing 12.5 g of NaOH in a 62.5 mL of ultrapure water. The solution was stirred at 60 °C for about 1 hour. In a separate flask, 25 g of glucose was dissolved into 37.5 mL of ultrapure water at 60 °C. The glucose solution was then added dropwise to the nitroisopthalic solution. Complete addition turned the nitroisopthalic solution to be dark brown. Afterwards, the nitroisopthalic solution was then cooled to room temperature air was bubbled through the solution for overnight. The solid obtained from this process was then filtered under reduced pressure and then dissolved in 100 mL of ultrapure water. This was followed by acidification by using 2 M of hydrochloric acid and was further acidified using 37% HCL until the pH was 1 to obtain an orange precipitate which was filtered and dried at 110 °C. The purity of the ligand was confirmed by H-NMR and C-NMR spectrum as can be seen in Figs S1 and S2, respectively.

### Synthesis of JUC-62

JUC-62 was synthesized according to the previously reported method^65^. In a typical synthesis, 0.24 g of copper nitrate trihydrate and 0.18 g of H_4_ABTC was firstly suspended in a mixture of DMF:ethanol:water (25 mL:15 mL:5 mL). It was observed that the starting material were not completely soluble in this mixture and resulted in a rather cloudy solution. Therefore, 2 M of nitric acid was then added dropwise to solubilize all of the starting material. Each drop of nitric acid added was followed by vigorous shaking. This was done in order to avoid adding too much nitric acid that would produce larger particle size. Addition was completed once a clear solution was obtained. The resulting solution was then put in an oven at 60 °C for 2 days. The resulting green product was then recovered by filtration under reduced pressure and stored in acetone for solvent exchange. Before membrane fabrication, the JUC-62 was filtered from acetone and then activated at 110 °C under vacuum. The JUC-62 data for nitrogen sorption, CO_2_ adsorption and CO_2_ dynamic photoswitching can be seen in Figures S4, S5 and S6, respectively.

### Synthesis of PCN-250

PCN-250 was synthesized according to the previously reported^41,66^ method with a slight modification to adjust the particle size. Before the MOF synthesis, a metal cluster from iron and cobalt was firstly synthesized. In a typical synthesis, 8 g of iron (III) nitrate hexahydrate and 18.3 g of cobalt (II) nitrate hexahydrate was dissolved in 70 mL of ultrapure water. In a separate flask, 4.4 M of sodium acetate trihydrate (70 mL solution) was also prepared. Afterwards, the sodium acetate trihydrate solution was poured into the metal solution to give a brown precipitate. The solution was kept overnight to continue the reaction. The precipitate was filtered the next day and washed with ultrapure water and ethanol and dried at room temperature under vacuum. For MOF synthesis, 75 mg of metal cluster and 50 mg of H_4_ABTC was dissolved in a mixture of 13.5 mL of DMF and 1.5 mL of acetic acid and heated in an oven at 140 °C for 12 hour. The resulting brown product was then filtered under reduced pressure and washed with DMF. This was followed by storing in DMF for about two days to remove the residual unreacted ligand and metal clusters. Afterwards, solvent exchange procedure took place where methanol was used to exchange the DMF. During this period, the MOF was immersed in methanol at 65 °C for 3 days. The last step was to exchange the methanol with dichloromethane. The MOF immersed in dichloromethane was put in the stainless steel autoclave and kept at 60 °C for about 3 days. Before membrane fabrication, the PCN-250 was activated at 190 °C under vacuum. Together with JUC-62 data, the PCN-250 data for nitrogen sorption, CO_2_ adsorption and CO_2_ dynamic photoswitching can be seen in Figs S4, S5 and S6, respectively.

### Fabrication of mixed matrix membrane

**Figure 8:**
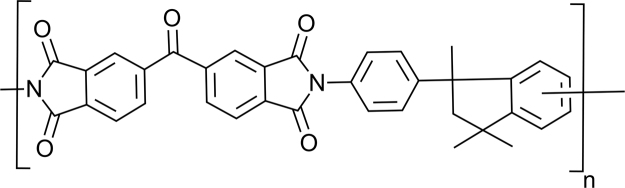
Structure of matrimid.

Mixed matrix membrane was fabricated by varying the particle loading inside the polymer matrix. Both JUC-62 and PCN-250 were used as the investigated incorporated-particles. Meanwhile, ZIF-8 (BASF) was also used as the incorporated particle as a control experiment. Matrimid was used as the polymer matrix and its structure is given in Fig. 8.

The mixed matrix membranes were fabricated by adding step-by-step all the required polymer into the MOF-suspension. In a typical synthesis that contains 0.5 g of total solids (MOFs and polymer), the required amount of particles (ZIF-8, JUC-62 and PCN-250) were firstly dispersed in dichloromethane. This was followed by dissolving about 30% of the total required polymer to the suspension and the solution was kept stirred overnight. Afterwards, another 30% of the required polymer was added and the solution was kept stirred for another 8 hours. The rest of the polymer was then added and the final suspension was kept stirred for overnight. The final suspension was then cast on a 7 cm petri dish which was covered by aluminium foil decorated with six 2 mm diameter holes. The membrane was left to dry in this condition by evaporating the solvent and followed by oven drying at 90 °C to completely remove all the remaining solvent inside the membrane. Once dried, the membrane was then cut to be around 25 mm in diameter and mounted on an aluminium washer which was glued with epoxy resin (See Figure S7 in supplementary information). This cutting process was to ensure that the membrane was located at the centre of the membrane testing cell and received maximum exposure from the UV light LED during UV light irradiation experiment (see the method for the UV-light gas permeation measurement).

It was observed that the highest loading that could be obtained from JUC-62 and PCN-250-matrimid mixed matrix membrane was 15 and 10 wt%, respectively. Above that, the structure of the membrane would be too brittle and also resulted in a defective membrane.

### General gas permeation testing apparatus

Gas permeation of the membranes was tested by using constant-volume variable-pressure approach (see Figure S9 for the general set up). The membrane area tested was around 4 cm^2^. The upstream pressure for both CO_2_ and N_2_ were maintained at 4 bar during the whole experiment. The temperature of the experiment was recorded to be around 37 °C using a thermocouple located at the centre inside the membrane testing cell (see Figure S11 in supplementary information). The membrane thickness was measured using a digital calliper. For the pristine matrimid, the membrane thickness was found to be around 50–60 µm while the mixed matrix membrane varied between 80–100 µm.

### Light-responsive gas permeation testing

**Figure 9:**
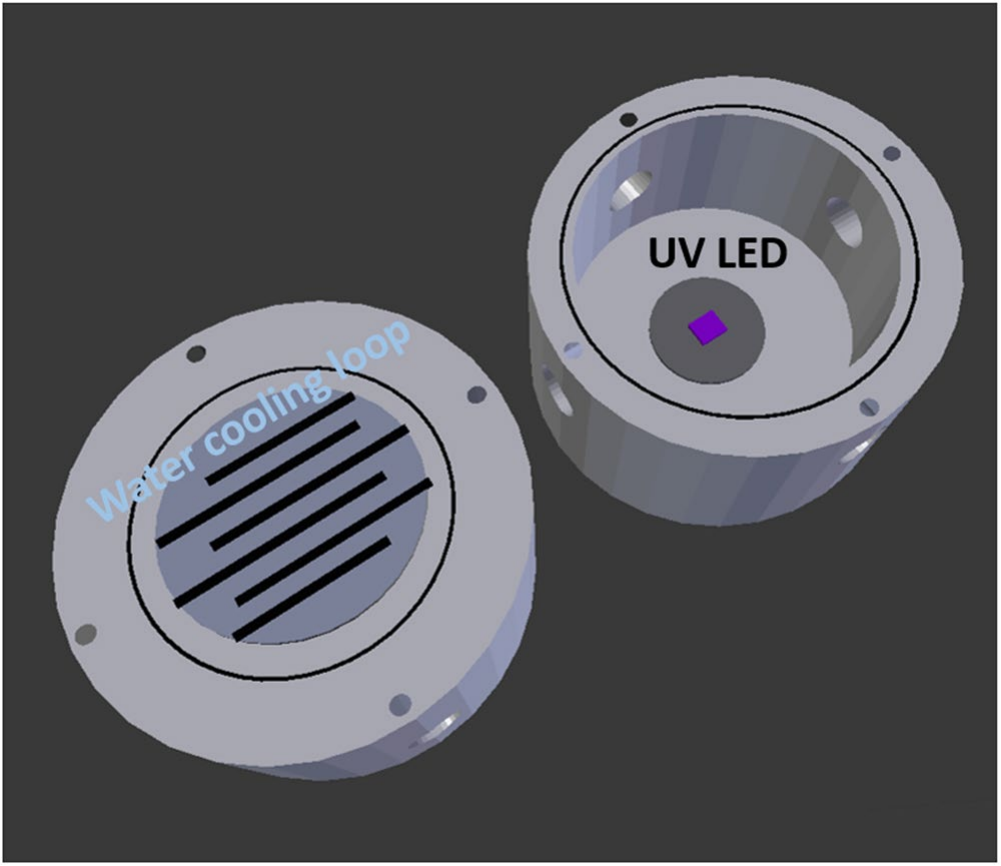
Schematic figure of the custom-made membrane test cell.

In order to evaluate the light-responsive ability of the resulting mixed-matrix membranes, we used our custom-designed membrane testing cell (see Figure S8 for the technical drawing and S10 when it was in operation). This membrane testing cell was also used during the gas permeation testing in the absence of UV light. Figure 9 gives a representation of the membrane test cell used for this study. A more detail and complete information about the membrane test cell dimension is provided in supplementary information (Section 3 supplementary information). First of all, it could be seen from the figure that there are two important features in this particular membrane testing cell: *in-situ* UV light and water loop system. Both features will be discussed in more detail below.

The presence of UV-light inside the membrane testing cell ensured that there would be sufficient light coming to the membrane without having to use external light source. In more details, the 365 nm UV light used was purchased from Thorlabs and it was mounted by using epoxy resin on the inner part of the membrane testing cell. The diameter of the whole piece of UV LED (including the PCB) was 22 mm. As described previously, the membrane was also cut around this size in order to maximize the exposure of the membrane with the UV light. During the experiment, the UV LED was connected with a power source that was maintained at 3.63 V and 0.890 A. Apart from contributing in giving consistent irradiation, maintaining the power level of the UV light was also important to maintain the temperature during the experiment which was further controlled using the water loop system as discussed below.

As previously stated, the function of the water cooling loop system is mainly to control the temperature inside the membrane testing cell during the experiment. This is particularly important since when the UV light was turned on, it generated a significant amount of heat which would significantly affect the measurement. Thus, a cooling off system was required. The water from temperature-controlled water bath was then flowed through the system during the whole experiment (both UV on and off). A different scenario happened when the UV light was off. In this case, there is no heat generated inside the cell and thus the main function of the water loop system is to maintain the temperature inside the cell to be more or less the same with the temperature when the UV light was on. Having said that, when the UV light was turned off, the water bath temperature was maintained at 42 °C and during the UV light exposure experiment, the water bath temperature was maintained at 31 °C. This system had successfully maintained the temperature inside the membrane cell to be around 37 °C for the whole experiment.

### Characterization techniques

#### X-Ray Diffraction

PXRD spectrum was collected using PAN-analytical instrument using Cu-Kα as the X-Ray source. The voltage and current was maintained at 40 kV and 20 mA, respectively. The spectrum was collected between from 5–30 in 2 theta. The 2theta scan step for all samples was 0.008°. For the MOF powder, about 20 mg of sample was used without grinding. For the mixed matrix membranes, the film was cut to be around 1 cm in diameter and then mounted on the sample holder. The sample was let to spin during the measurement.

#### Fourier Transformed Infrared Spectroscopy (FTIR)

FTIR of the samples were collected using ATR technique. The equipment used was Perkin-Elmer Spectrum 100 ATR-FTIR Spectrometer. For irradiated sample, the mixed matrix membranes sample were first irradiated for about 5 minutes using Omnicure S1500 and then immediately followed by measuring the spectrum.

#### Thermogravimetric analysis (TGA)

TGA analysis was conducted using Netzsch STA 449 F5 Jupiter apparatus. About 20 mg of sample was used both for MOF particle and mixed matrix membranes. For the membranes, the film was cut into small pieces before putting into the sample pan. The heating rate was set to 10 °C.min^−1^ and the sample was measured under nitrogen atmosphere flowing at 20 mL min^−1^.

#### Scanning Electron Microscopy (SEM)

SEM characterization was carried out using a field emission gun scanning electron microscope (FEGSEM) LEO Gemini 1525. The sample was sputtered with gold to increase conductivity prior to imaging.

### Data availability

All data for this study is freely available from the following repository: 10.5281/zenodo.1040278.

## Electronic supplementary material

Supplementary Information

## References

[1] Bryjak M, Wolska J, Siekierka A, Kujawski J (2015). Stimuli responsive membranes in separation processes-short review. Copernican Letters.

[2] Wesson, R. D., Dow, E. S. & Williams, S. R. Responsive Membranes/Material‐Based Separations: Research and Development Needs. *Responsive Membranes and Materials* 385–393 (2012).

[3] Park CH (2016). Mixed matrix membranes containing functionalized multiwalled carbon nanotubes: Mesoscale simulation and experimental approach for optimizing dispersion. Journal of Membrane Science.

[4] Lin R, Ge L, Diao H, Rudolph V, Zhu Z (2016). Propylene/propane selective mixed matrix membranes with grape-branched MOF/CNT filler. Journal of Materials Chemistry A.

[5] Shirzadeh-Gharacheh A, Rahbari-Sisakht M (2016). Polyvinylidene fluoride hollow fiber mixed matrix membrane contactor incorporating modified ZSM-5 zeolite for carbon dioxide absorption. RSC Advances.

[6] Zarshenas K, Raisi A, Aroujalian A (2016). Mixed matrix membrane of nano-zeolite NaX/poly (ether-block-amide) for gas separation applications. Journal of Membrane Science.

[7] Erucar I, Yilmaz G, Keskin S (2013). Recent Advances in Metal–Organic Framework‐Based Mixed Matrix Membranes. Chemistry–An Asian Journal.

[8] Campbell J, Székely G, Davies R, Braddock DC, Livingston AG (2014). Fabrication of hybrid polymer/metal organic framework membranes: mixed matrix membranes versus *in situ* growth. Journal of Materials Chemistry A.

[9] Kang Z (2015). Mixed matrix membranes composed of two-dimensional metal–organic framework nanosheets for pre-combustion CO 2 capture: a relationship study of filler morphology versus membrane performance. Journal of Materials Chemistry A.

[10] Dong G, Li H, Chen V (2013). Challenges and opportunities for mixed-matrix membranes for gas separation. Journal of Materials Chemistry A.

[11] Gu, Z.-G. & Zhang, J. Epitaxial growth and applications of oriented metal–organic framework thin films. *Coord. Chem. Rev*. (2017).

[12] Gu Z-G (2015). Transparent films of metal-organic frameworks for optical applications. Microporous Mesoporous Mater..

[13] Gu ZG (2017). MOF‐Templated Synthesis of Ultrasmall Photoluminescent Carbon‐Nanodot Arrays for Optical Applications. Angew. Chem. Int. Ed..

[14] Dybtsev DN, Chun H, Kim K (2004). Rigid and Flexible: A Highly Porous Metal–Organic Framework with Unusual Guest‐Dependent Dynamic Behavior. Angew. Chem. Int. Ed..

[15] Schaber J (2017). *In Situ* Monitoring of Unique Switching Transitions in the Pressure-Amplifying Flexible Framework Material DUT-49 by High-Pressure 129Xe NMR Spectroscopy. The Journal of Physical Chemistry C.

[16] Zhao D, Yuan D, Krishna R, van Baten JM, Zhou H-C (2010). Thermosensitive gating effect and selective gas adsorption in a porous coordination nanocage. Chem. Commun..

[17] Li, H. *et al*. Magnetic induction framework synthesis: a general route to the controlled growth of metal-organic frameworks. *Chem. Mater*. (2017).

[18] Mukhopadhyay RD, Praveen VK, Ajayaghosh A (2014). Photoresponsive metal–organic materials: exploiting the azobenzene switch. Materials Horizons.

[19] Song, W.-C., Cui, X.-Z., Liu, Z.-Y., Yang, E.-C. & Zhao, X.-J. Light-triggered Supramolecular Isomerism in a Self-catenated Zn (II)-organic Framework: Dynamic Photo-switching CO_2_ Uptake and Detection of Nitroaromatics. *Scientific reports* **6** (2016). 10.1038/srep34870 PMC5057147 27725711

[20] Brown JW (2013). Photophysical pore control in an azobenzene-containing metal–organic framework. Chemical Science.

[21] Yanai N (2012). Guest-to-host transmission of structural changes for stimuli-responsive adsorption property. J. Am. Chem. Soc..

[22] Park J (2011). Reversible alteration of CO2 adsorption upon photochemical or thermal treatment in a metal–organic framework. J. Am. Chem. Soc..

[23] Modrow A, Zargarani D, Herges R, Stock N (2011). The first porous MOF with photoswitchable linker molecules. Dalton Transactions.

[24] Huang H, Sato H, Aida T (2017). Crystalline Nanochannels with Pendant Azobenzene Groups: Steric or Polar Effects on Gas Adsorption and Diffusion?. J. Am. Chem. Soc..

[25] Castellanos, S. *et al*. Structural Effects in Visible‐Light‐Responsive Metal–Organic Frameworks Incorporating ortho‐Fluoroazobenzenes. *Chemistry–A European Journal* (2015). 10.1002/chem.201503503 26617393

[26] Luo F (2014). Photoswitching CO2 Capture and Release in a Photochromic Diarylethene Metal–Organic Framework. Angew. Chem. Int. Ed..

[27] Li H (2016). MaLISA–a cooperative method to release adsorbed gases from metal–organic frameworks. Journal of Materials Chemistry A.

[28] Lyndon R (2013). Dynamic Photo‐Switching in Metal–Organic Frameworks as a Route to Low‐Energy Carbon Dioxide Capture and Release. Angew. Chem. Int. Ed..

[29] Prasetya N, Ladewig BP (2017). Dynamic photo-switching in light-responsive JUC-62 for CO 2 capture. Scientific Reports.

[30] Ladewig, B., Lyndon, R. & Hill, M. Gas Separation Processes. Patent Application WO2014015383A1 (2014).

[31] Knebel A (2017). Azobenzene Guest Molecules as Light-Switchable CO2 Valves in an Ultrathin UiO-67 Membrane. Chem. Mater..

[32] Wang Z (2016). Tunable molecular separation by nanoporous membranes. Nature communications.

[33] Fu W-Q, Liu M, Gu Z-G, Chen S-M, Zhang J (2016). Liquid Phase Epitaxial Growth and Optical Properties of Photochromic Guest-Encapsulated MOF Thin Film. Crystal Growth & Design.

[34] Gu Z-G, Chen Z, Fu W-Q, Wang F, Zhang J (2015). Liquid-Phase Epitaxy Effective Encapsulation of Lanthanide Coordination Compounds into MOF Film with Homogeneous and Tunable White-Light Emission. ACS applied materials & interfaces.

[35] Valero M, Zornoza B, Téllez C, Coronas J (2014). Mixed matrix membranes for gas separation by combination of silica MCM-41 and MOF NH 2-MIL-53 (Al) in glassy polymers. Microporous Mesoporous Mater..

[36] Tien-Binh N, Vinh-Thang H, Chen XY, Rodrigue D, Kaliaguine S (2016). Crosslinked MOF-polymer to enhance gas separation of mixed matrix membranes. Journal of Membrane Science.

[37] Wu X (2018). Nanoporous ZIF-67 embedded polymers of intrinsic microporosity membranes with enhanced gas separation performance. Journal of Membrane Science.

[38] Chen C-J, Liu G-Y, Liu X-S, Li D-D, Ji J (2012). Construction of photo-responsive micelles from azobenzene-modified hyperbranched polyphosphates and study of their reversible self-assembly and disassembly behaviours. New Journal of Chemistry.

[39] Jacob H (2014). Monitoring the reversible photoisomerization of an azobenzene-functionalized molecular triazatriangulene platform on Au (111) by IRRAS. Physical Chemistry Chemical Physics.

[40] Yi Q, Sukhorukov GB (2014). UV-induced disruption of microcapsules with azobenzene groups. Soft Matter.

[41] Li, H. *et al*. A robust metal organic framework for dynamic light‐induced swing adsorption of carbon dioxide. *Chemistry-A European Journal* (2016). 10.1002/chem.201602671 27273621

[42] Zhao H-Y, Cao Y-M, Ding X-L, Zhou M-Q, Yuan Q (2008). Effects of cross-linkers with different molecular weights in cross-linked Matrimid 5218 and test temperature on gas transport properties. Journal of Membrane Science.

[43] Becker D, Konnertz N, Böhning M, Schmidt J, Thomas A (2016). Light-switchable polymers of intrinsic microporosity. Chem. Mater..

[44] Song Q (2012). Zeolitic imidazolate framework (*Z*IF-8) based polymer nanocomposite membranes for gas separation. Energy & Environmental Science.

[45] Li H, Hill MR (2017). Low-Energy CO2 Release from Metal–Organic Frameworks Triggered by External Stimuli. Acc. Chem. Res..

[46] Hsieh JO, Balkus KJ, Ferraris JP, Musselman IH (2014). MIL-53 frameworks in mixed-matrix membranes. Microporous Mesoporous Mater..

[47] Sumer, Z. & Keskin, S. Computational Screening of MOF-Based Mixed Matrix Membranes for CO_2_/N_2_ Separations. *Journal of Nanomaterials* **2016** (2016).

[48] Khan AL, Klaysom C, Gahlaut A, Khan AU, Vankelecom IF (2013). Mixed matrix membranes comprising of Matrimid and–SO 3 H functionalized mesoporous MCM-41 for gas separation. Journal of membrane science.

[49] Perez EV, Balkus KJ, Ferraris JP, Musselman IH (2009). Mixed-matrix membranes containing MOF-5 for gas separations. Journal of Membrane Science.

[50] Venna SR (2015). Fabrication of MMMs with improved gas separation properties using externally-functionalized MOF particles. Journal of Materials Chemistry A.

[51] Zhang Y, Musselman IH, Ferraris JP, Balkus KJ (2008). Gas permeability properties of Matrimid® membranes containing the metal-organic framework Cu–BPY–HFS. Journal of Membrane Science.

[52] Musselman, I., Balkus, K. Jr & Ferraris, J. Mixed-Matric Membranes for CO_2_ and H_2_ Gas Separations Using Metal-Organic Framework and Mesoporus Hybrid Silicas. (University Of Texas At Dallas, 2009).

[53] Dechnik J, Sumby CJ, Janiak C (2017). Enhancing Mixed-Matrix Membrane Performance with Metal-Organic Framework Additives. Cryst. Growth Des.

[54] Arab P, Parrish E, İslamoğlu T, El-Kaderi HM (2015). Synthesis and evaluation of porous azo-linked polymers for carbon dioxide capture and separation. Journal of Materials Chemistry A.

[55] Yang Z (2015). Azo-functionalized microporous organic polymers: synthesis and applications in CO 2 capture and conversion. Chem. Commun..

[56] Patel HA (2014). Directing the Structural Features of N2‐Phobic Nanoporous Covalent Organic Polymers for CO2 Capture and Separation. Chemistry-A European Journal.

[57] Patel HA (2013). Unprecedented high-temperature CO2 selectivity in N2-phobic nanoporous covalent organic polymers. Nature communications.

[58] Zhang H (2016). Microporous organic polymers based on tetraethynyl building blocks with N-functionalized pore surfaces: synthesis, porosity and carbon dioxide sorption. RSC Advances.

[59] Zhao S (2016). Channel-wall functionalization in covalent organic frameworks for the enhancement of CO 2 uptake and CO 2/N 2 selectivity. RSC Advances.

[60] Buyukcakir O (2015). Systematic Investigation of the Effect of Polymerization Routes on the Gas‐Sorption Properties of Nanoporous Azobenzene Polymers. Chemistry-A European Journal.

[61] Nagaraja C, Haldar R, Maji TK, Rao C (2012). Chiral porous metal–organic frameworks of Co (II) and Ni (II): synthesis, structure, magnetic properties, and CO2 uptake. Crystal Growth & Design.

[62] Lau CH (2014). Ending aging in super glassy polymer membranes. Angew. Chem. Int. Ed..

[63] Smith, S. J., Ladewig, B. P., Hill, A. J., Lau, C. H. & Hill, M. R. Post-synthetic Ti exchanged UiO-66 metal-organic frameworks that deliver exceptional gas permeability in mixed matrix membranes. *Scientific reports* **5** (2015). 10.1038/srep07823 PMC4296298 25592747

[64] Ameerunisha S, Zacharias PS (1995). Characterization of simple photoresponsive systems and their applications to metal ion transport. Journal of the Chemical Society, Perkin Transactions.

[65] Wu, Y. *et al*. Determination of Hg (II) in tea and mushroom samples based on metal-organic frameworks as solid phase extraction sorbents. *Microporous Mesoporous Mater*. 204–210 (2016).

[66] Feng, D. *et al*. Kinetically tuned dimensional augmentation as a versatile synthetic route towards robust metal–organic frameworks. *Nature communications* **5** (2014). 10.1038/ncomms6723 25474702

