# Supplementary material for: Matrimid-JUC-62 and Matrimid-PCN-250 mixed matrix membranes displaying light-responsive gas separation and beneficial ageing characteristics for CO2/N2 separation
Source: Sci Rep. 2018 Feb 13;8:2944. doi: 10.1038/s41598-018-21263-7 (PMC5811445; doi:10.1038/s41598-018-21263-7)
Supplement: Supplementary file 1 — Supplementary Information [file 41598_2018_21263_MOESM1_ESM.docx]

**Electronic Supplementary Information**

**for**

**Matrimid-JUC-62 and Matrimid-PCN-250 mixed matrix membranes displaying light-responsive gas separation and beneficial ageing characteristics for CO2/N2 separation**

Nicholaus Prasetya,^a^ Anastasia Teck^a^ and Bradley P. Ladewig^a, †^

^a^Barrer Centre, Department of Chemical Engineering, Imperial College London, Exhibition Road, London SW7 2AZ, United Kingdom

^†^Corresponding author: Bradley P. Ladewig, email: [b.ladewig@imperial.ac.uk](mailto:b.ladewig@imperial.ac.uk)

1. **Ligand Purity**

Ligand purity was checked by using H-NMR and C-NMR. About 20 mg of sample was dissolved in about 7 mL of d6-DMSO. NMR spectrum was collected by using Bruker Av-400 instrument. The result for H-NMR and C-NMR are given in Figure S1 and S2, respectively.

|  |
| --- |

Figure S 1. H-NMR spectrum of 3,3’-5,5’-azobenzene tetracarboxylic acid (d6-DMSO) (^1^H NMR (400 MHz, DMSO) δ 8.61-8.57 (t, J = 1.6 Hz, 2H), 8.56-8.54 (d, J = 1.6 Hz, 4H))


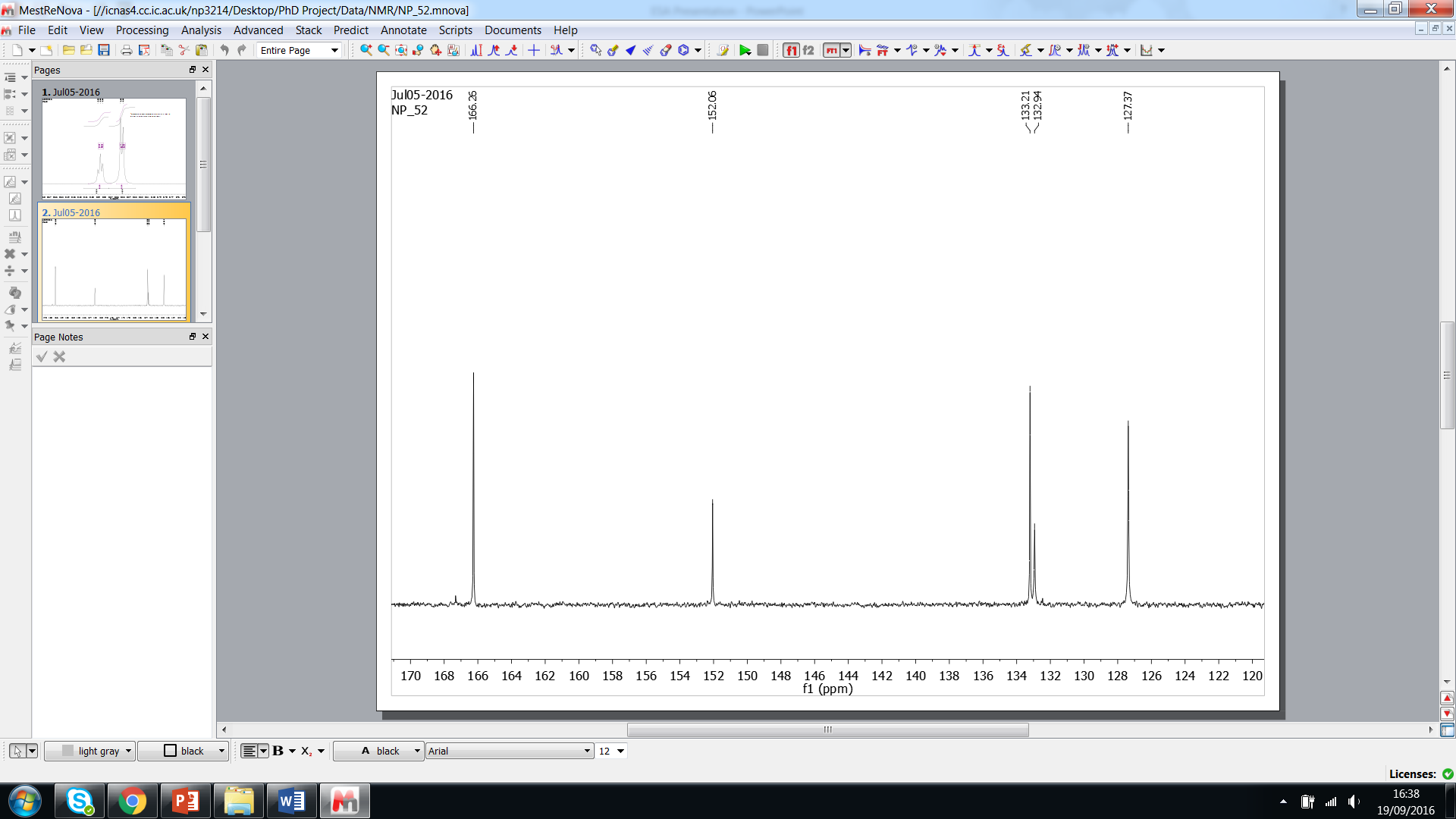


Figure S 2 C-NMR Spectrum of 3,3’-5,5’-azobenzene tetracarboxylic acid (^13^C NMR (101 MHz, DMSO) δ 166.26, 152.06, 133.21, 132.94, 127.37)

1. **Crystal Characterization**

**2.1 Crystal Structure**

Crystal structure pictures were generated using Mercury software

CCDC number for JUC-62: 666395, code for Mercury: OFOCUI

CCDC number for PCN-250: 975784, code for Mercury: TOWPEC

| 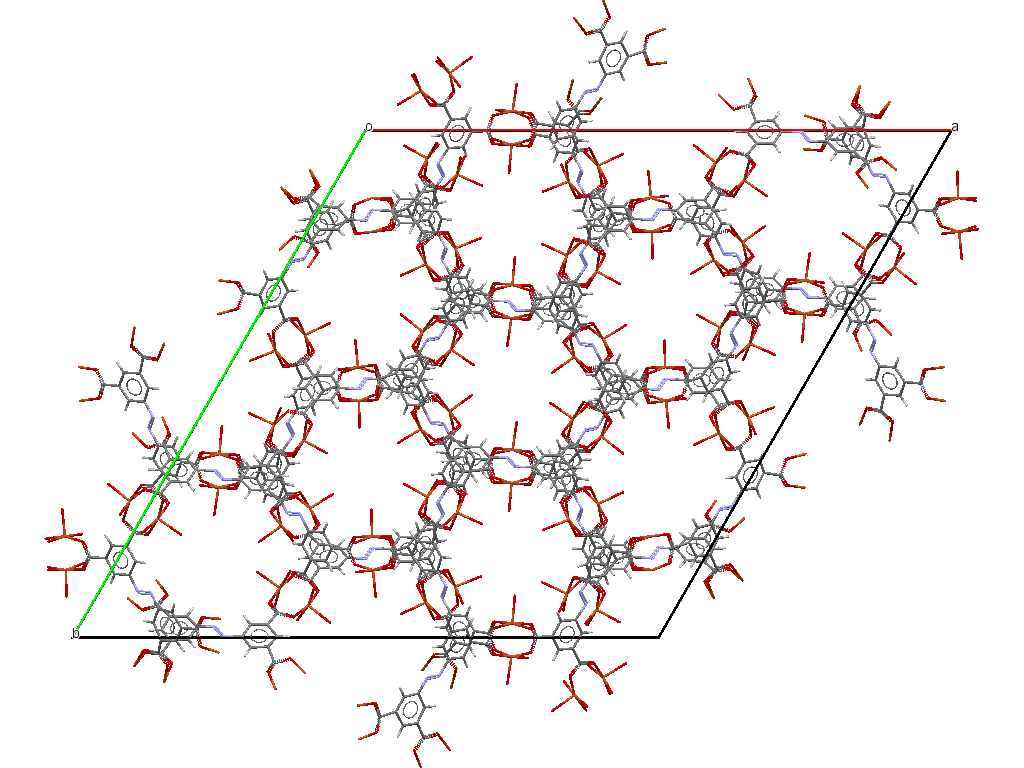 |
| --- |
| (a) |
| 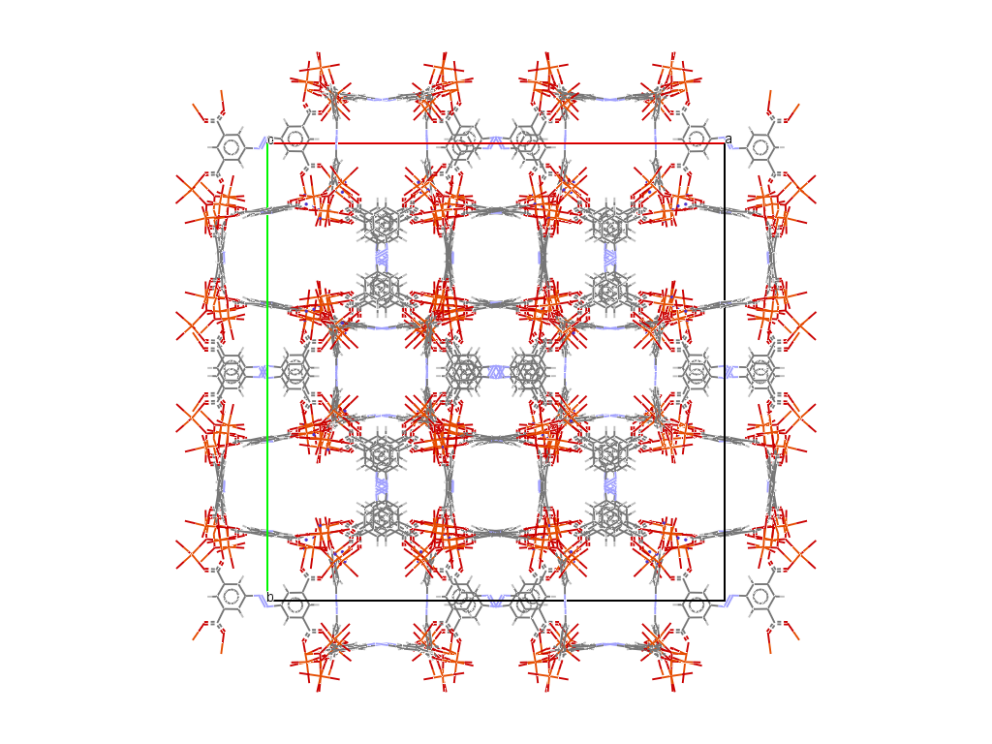 |
| (b) |

Figure S 3. Crystal structure of JUC-62 (A) and PCN-250 (B) used in the study viewed along c axis

**2.2 Nitrogen sorption**

Apart from PXRD, we also checked the nitrogen sorption for both crystals. Nitrogen sorption of both JUC-62 and PCN-250 was measured using Micromeritic Tristar instrument. The measurement was taken at 77 K. The BET surface area calculated for JUC-62 and PCN-250 was found to be 1019 ± 20 m2/g and 1376 ± 27 m^2^/g, respectively.

**
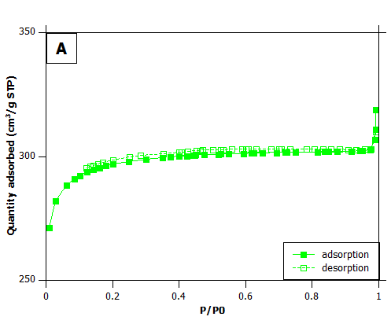

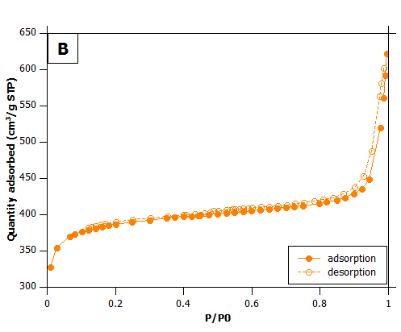
**

Figure S 4. Nitrogen sorption isotherm of JUC-62 (A) and PCn-250 (B)

**2.3 CO_2_ adsorption**

CO_2_ adsorption was also measured at 0^o^C to observe its carbon dioxide capacity uptake. Micromeritic 3Flex was used as the main equipment and the surrounding temperature was controlled by using ISO Controller. The result is given in Figure S5.

**
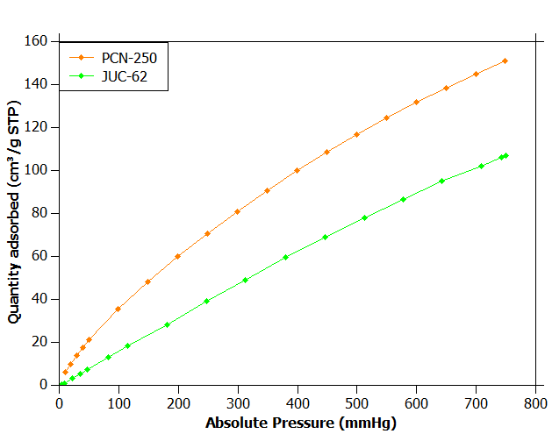
**

Figure S 5. CO2 adsorption of JUC-62 and PCN-250 at 0^o^C

Lastly, the crystals were also checked regarding its photo-responsive ability. The details of such measurement has been explained in details previously^1^.

**
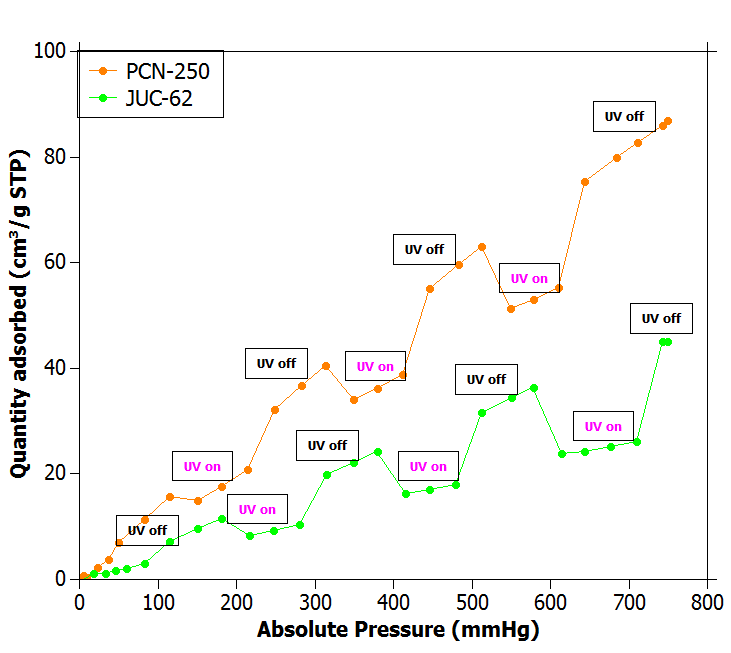
**

Figure S 6. CO_2_ photoswitching of JUC-62 and PCN-250 at 25^o^C

1. **Membrane Separation Experimental Details**

**3.1 Membrane fabrication**

**
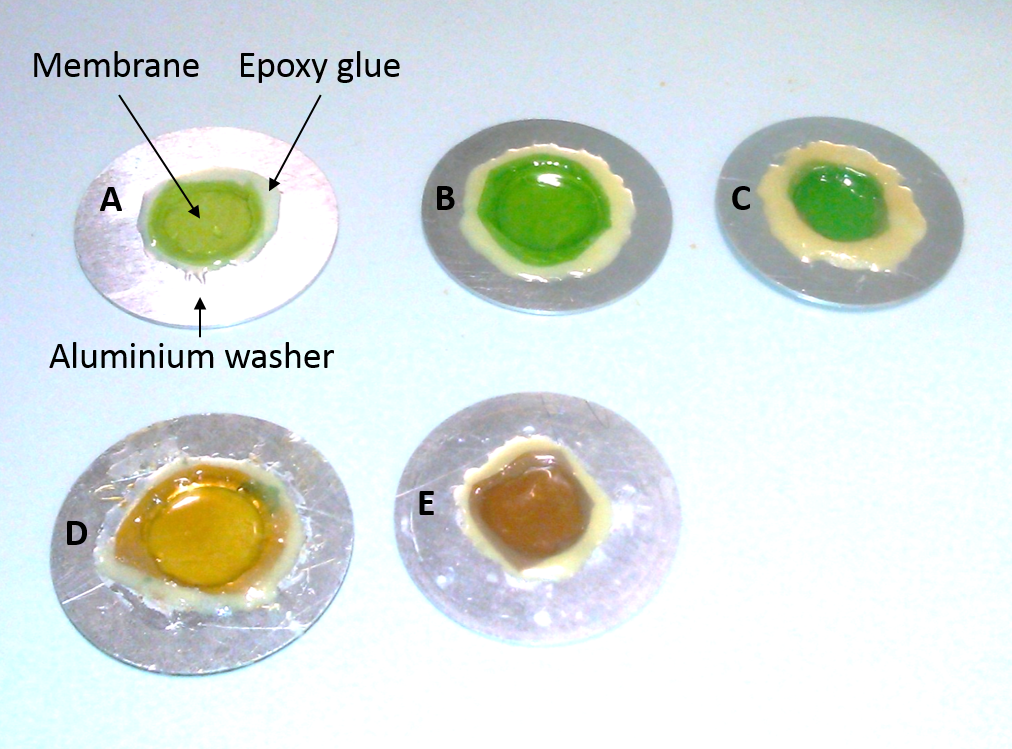
**

Figure S 7. Picture of membranes used in the study: 5 wt% JUC-62-matrimid (A), 10 wt% JUC-62-matrimid (B), 15 wt% JUC-62-matrimid (C), 5 wt% PCN-250-matrimid (D) and 10 wt% PCN-250-matrimid (E)

There were five different mixed matrix membranes used in this study as shown in the picture: (A) 5 wt% JUC-62-matrimid, (B) 10 wt% JUC-62-matrimid, (C) 15 wt% JUC-62-matrimid, (D) 5 wt% PCN-250-matrimid and (E) 10 wt% PCN-250-matrimid. As stated in the main manuscript, the membranes were cut into approximately 25 mm in diameter and then mounted on an aluminium washer using epoxy glue as shown above. This would ensure that the membranes got focused exposure from the UV light mounted inside the cell since the diameter of the UV LED was 22 mm (see the complete technical drawing in the figure below)

**3.2 Custom-made membrane testing cell technical drawing**

Figure S 8. Technical drawing of the custom-made membrane testing cell

The figure S8 below gives the technical drawing of our custom-made membrane testing cell, including the detail how we designed the water loop cooling system on top of the membrane testing cell so it would be efficient in taking up heat generated from the UV light.

**3.3 Experimental set-up and details**

The whole experimental set up and how the UV light operating inside the membrane test cell was given in Figure S9 and S10, respectively.

**
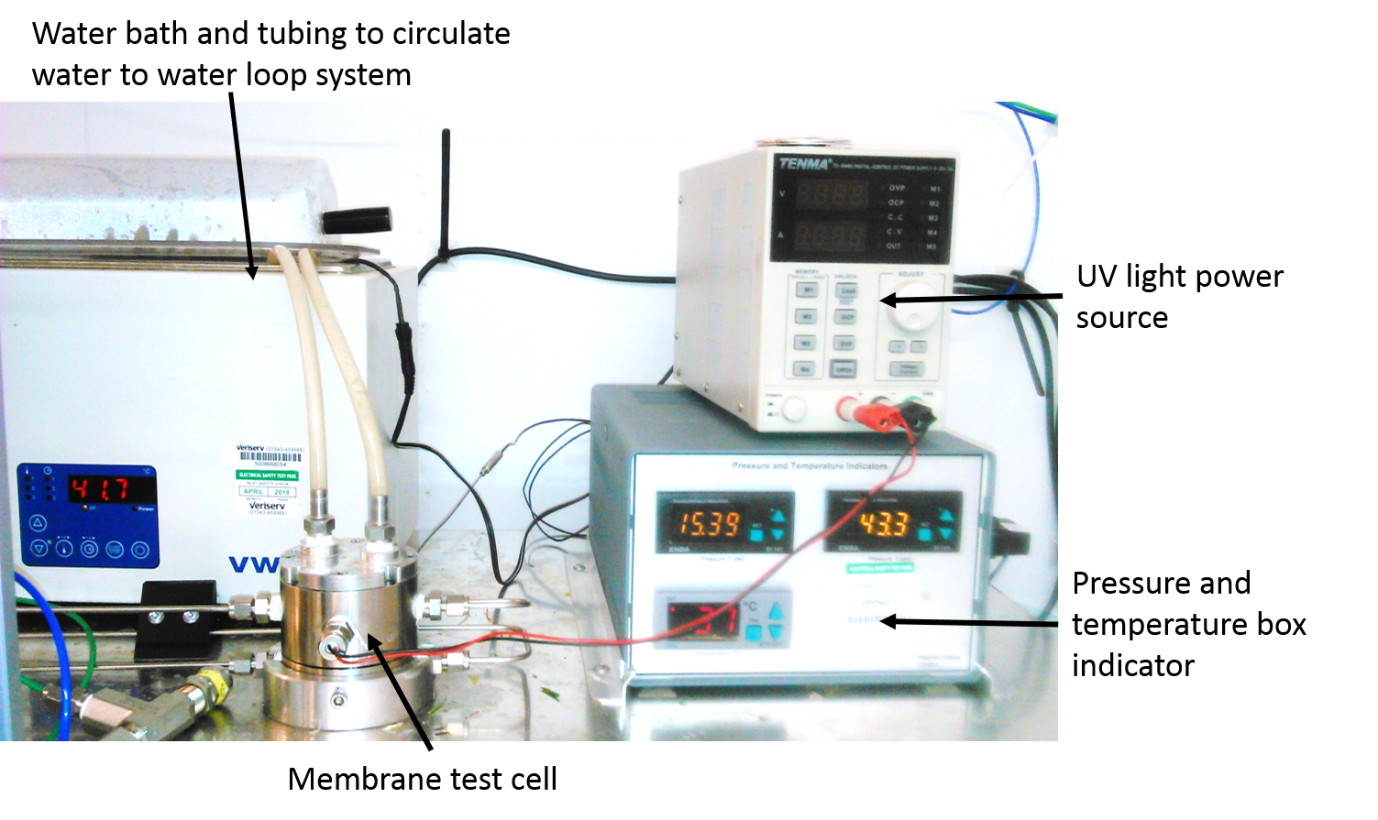
**

Figure S 9. Picture of the whole experimental set-up

**
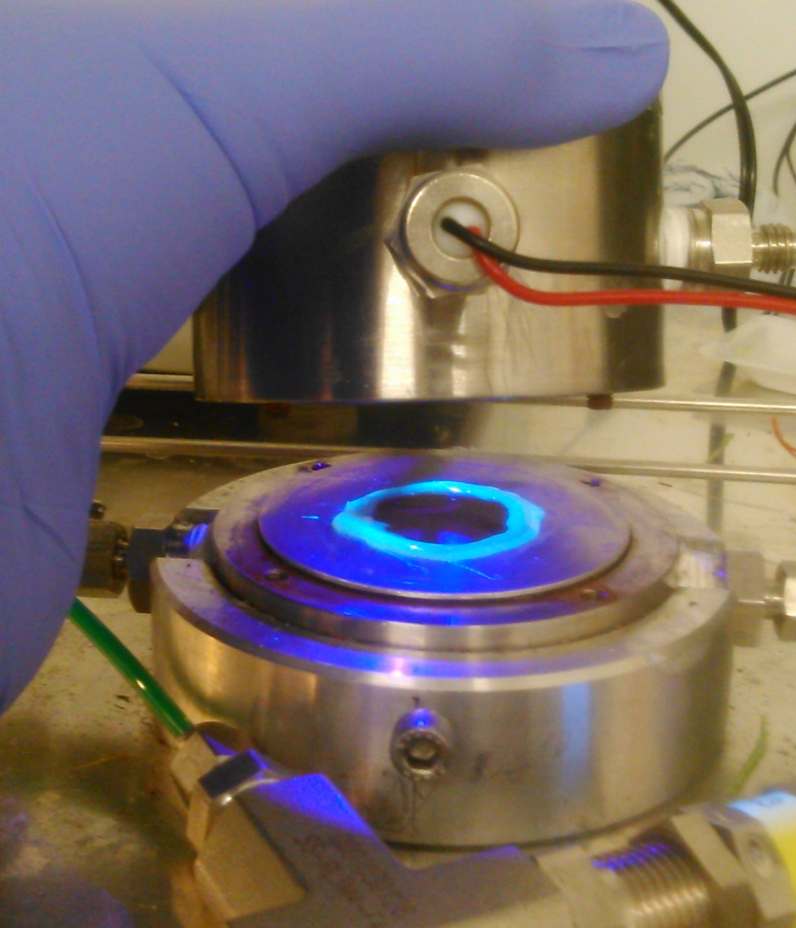
**

Figure S 10. Picture of the inside of the custom-made membrane testing cell with UV light on

As can be seen from the figure, when the UV LED was switched on, all the light were focused on the membrane mounted onto the washer.

As described in the main manuscript, regulating the temperature between the period of UV light switching is important. A typical temperature graph ws given below showing that there was negligible change in temperature during the experiment. This also explains why there is no change in membrane permeability for the control experiment.

**
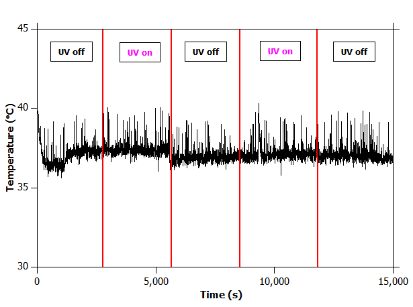
**

Figure S 11. Temperature profile during the experiment

1. **Membrane performance supporting information**

**4.1 Additional FTIR Spectrum**

**

**

Figure S 12. FTIR spectrum of 15 wt% JUC-62-matrimid (A) and 10 wt% PCN-250-matrimid (B) measured under normal and UV-irradiation

It could be seen from the figure below that there was only a slight change in FTIR spectrum between non-irradiated and irradiated mixed matrix membranes. This might partially explain why we only observed a modest degree of photoswitching in the mixed matrix membranes.

**4.2 Nitrogen photoswitching experiment**

**
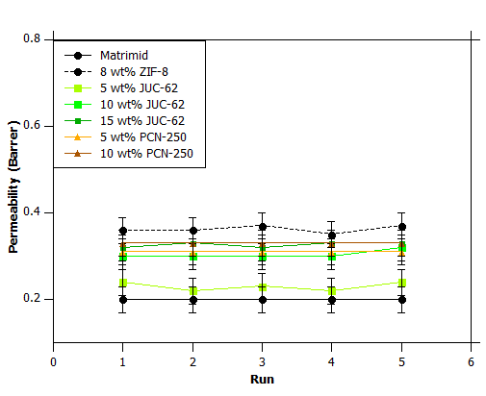
**

Figure S 13. Nitrogen photoswitching experiment

Apart from CO_2_ permeability photoswitching, we also conducted nitrogen permeability photoswitching. However, since the permeability of the nitrogen for all the membranes tested were quite low, we did not observed any switching behaviour for nitrogen. We did see a slight fluctuations but in general they were still in the error range.

**4.3 Performance comparison**

Figure S 14. CO_2_/N_2_ ideal selectivity comparison of our mixed matrix membranes and the rest of MOF-matrimid mixed matrix membranes fabricated as dense film

This chart represents the improvement in CO_2_/N_2_ ideal selectivity in the MOF-matrimid dense mixed matrix membrane system. In order to make a fairer comparison, the chart here shows how much improvement (in percentage) were achieved for the reported membranes^2-5^ compared with their own matrimid membrane. To endure fairness, this study only compares MOF-matrimid mixed matrix membranes fabricated as dense membrane and only CO_2_/N_2_ ideal selectivity is considered for comparison. This would then eliminate the effect coming from various variables such as operating condition and intrinsic property of the matrimid.

**References**

1. N. Prasetya and B. P. Ladewig, *Scientific reports*, 2017, **7**, 13355.

2. A. L. Khan, C. Klaysom, A. Gahlaut, A. U. Khan and I. F. Vankelecom, *Journal of membrane science*, 2013, **447**, 73-79.

3. E. V. Perez, K. J. Balkus, J. P. Ferraris and I. H. Musselman, *Journal of Membrane Science*, 2009, **328**, 165-173.

4. Y. Zhang, I. H. Musselman, J. P. Ferraris and K. J. Balkus, *Journal of Membrane Science*, 2008, **313**, 170-181.

5. S. R. Venna, M. Lartey, T. Li, A. Spore, S. Kumar, H. B. Nulwala, D. R. Luebke, N. L. Rosi and E. Albenze, *Journal of Materials Chemistry A*, 2015, **3**, 5014-5022.
